# Clinical Prognostic Value of the *PLOD* Gene Family in Lung Adenocarcinoma

**DOI:** 10.3389/fmolb.2021.770729

**Published:** 2022-02-21

**Authors:** Yiming Meng, Jing Sun, Guirong Zhang, Tao Yu, Haozhe Piao

**Affiliations:** ^1^ Department of Central Laboratory, Cancer Hospital of China Medical University, Liaoning Cancer Hospital and Institute, Shenyang, China; ^2^ Department of Biobank, Cancer Hospital of China Medical University, Liaoning Cancer Hospital and Institute, Shenyang, China; ^3^ Department of Medical Imaging, Cancer Hospital of China Medical University, Liaoning Cancer Hospital and Institute, Shenyang, China; ^4^ Department of Neurosurgery, Cancer Hospital of China Medical University, Liaoning Cancer Hospital and Institute, Shenyang, China

**Keywords:** PLOD family member, LUAD, diagnosis, prognosis, immune infiltration

## Abstract

Accumulating evidence has implicated members of the procollagen-lysine, 2-oxoglutarate 5-dioxygenase (PLOD) gene family, PLOD1, PLOD2, and PLOD3, in cancer progression and metastasis. However, their expression, prognostic value, and mechanisms underlying their roles in lung adenocarcinoma (LUAD) have not yet been reported. We downloaded PLOD data for LUAD and normal tissues from The Cancer Genome Atlas (TCGA). PLOD1-3 protein expression was evaluated using the Clinical Proteomics Tumor Analysis Consortium and Human Protein Atlas. Survival analysis was performed using the Kaplan–Meier method. A protein–protein interaction network was constructed using STRING software. The “ClusterProfiler” package was used for functional-enrichment analysis. The relationship between PLOD mRNA expression and immune infiltration was analyzed using the Tumor Immunity Assessment Resource and Tumor Immune System Interaction Database. The expression of PLODs in LUAD tissues was significantly upregulated compared with that in adjacent normal tissues. PLOD mRNA overexpression is associated with lymph node metastasis and high TNM staging. Receiver operating characteristic curve analysis showed that when the cut-off level was 6.073, the accuracy, sensitivity, and specificity of PLOD1 in distinguishing LUAD from adjacent controls were 84.4, 79.7, and 82.6%, respectively. The accuracy, sensitivity, and specificity of PLOD2 in distinguishing LUAD from adjacent controls were 81.0, 98.3, and 68.0%, respectively, at a cut-off value of 4.360. The accuracy, sensitivity, and specificity of PLOD3 in distinguishing LUAD from adjacent controls were 69.0, 86.4, and 52.0%, respectively, with a cut-off value of 5.499. Kaplan–Meier survival analysis demonstrated that LUAD patients with high PLODs had a worse prognosis than those with low PLODs. Correlation analysis showed that PLOD mRNA expression was related to immune infiltration and tumor purity. Upregulation of PLOD expression was significantly associated with poor survival and immune cell infiltration in LUAD. Our research shows that PLOD family members have potential as novel biomarkers for poor prognosis and as potential immunotherapy targets for LUAD.

## Introduction

Lung cancer is one of the most malignant tumors that threaten human lives and health. Statistics on the pathological classification of lung cancer show that the most important type is non-small cell lung cancer (NSCLC), accounting for approximately 80% of all lung cancers. Lung adenocarcinoma (LUAD) is a common clinical type of NSCLC (Shergalis et al., 2020; Li et al., 2021). Although some progress has been made in surgical resection, chemotherapy, and radiotherapy, the 5-year survival rate of patients with LUAD remains low. The main reasons for this may be immunosuppression, cell proliferation, distant metastases, and drug resistance (Chen et al., 2021). Therefore, there is an urgent need to identify new, early diagnostic biomarkers and therapeutic targets for LUAD.

The procollagen-lysine, 2-ketoglutarate 5-dioxygenase (*PLOD*) gene family includes three members, *PLOD*1, *PLOD*2, and *PLOD*3, located on chromosomes 1p, 3q, and 7q, respectively. Multiple studies have shown that *PLOD* genes play essential roles in the development and progression of cancer (Guo et al., 2021a). Other studies have shown that, compared with normal kidney tissue, the expression of *PLOD* genes is increased in hepatocellular carcinoma (HCC) and positively correlated with the prognosis of HCC patients (Yang et al., 2020). In addition, bioinformatics analysis has shown that compared with adjacent normal tissues, *PLOD* gene family members are expressed at higher levels in LUAD tissues. However, the expression and prognostic effects of *PLOD* family members in LUAD require further elucidation.

To the best of our knowledge, the prognostic value of *PLODs* in LUAD and their correlation with immune infiltration are still not fully understood. To test this hypothesis, we evaluated TCGA database data and performed other bioinformatics analyses of the prognostic role of the *PLOD* family in LUAD. We further investigated the correlation between *PLOD* and immune cell infiltration.

## Materials and Methods

### Datasets Obtained From the Cancer Genome Atlas (TGCA) Datasets

TCGA lung adenocarcinoma (TCGA disease code: LUAD) cohorts were downloaded from TCGA official website (https://portal.gdc.cancer.gov/) (Tomczak et al., 2015). The 33 enrolled cancer types contained at least five samples in the normal group. At last, the RNA-Seq gene expression data with workflow type of fragments per kilobase per million (FPKM) were subsequently transformed to transcripts per million (TPM) and log2 conversion for further study. Since all the data are available from TCGA, no approval by an ethics committee is required.

### RNA Sequencing (RNA-Seq) Data of *PLOD*s in LUAD

Expression of *PLODs* in the RNA-Seq expression data in LUAD were also obtained from TCGA. This included 59 normal lung samples (normal-adjacent tissue to LUAD) and 535 LUAD tissue data. Specimen characteristics and associated clinical information including age, gender, smoker condition, tumor stage, and location are described. The mRNA expression data were summarized with means ± SD.

### Clinical Proteomic Tumor Analysis Consortium (CPTAC)

The protein expression profile was downloaded from the CPTAC resource (https://cptac-data-portal.georgetown.edu) and the proteome of each tumor sample characterized (Edwards et al., 2015). UALCAN (Chandrashekar et al., 2017) is a user-friendly online web resource. The R package “Seurat” was applied for data standardization. Then, the plots were drawn with the given cell classification using R package “ggplot2”. In this study, a throughput analysis of *PLOD* protein expression was presented.

### Biopsy Immunohistochemical Micrographs From the Human Protein Atlas (HPA)

The HPA (https://www.proteinatlas.org/) provides a map showing the distribution and relative abundance of human genes on protein level in normal human tissues and LUAD tissues (Uhlen et al., 2015; Uhlen et al., 2017). The HPA was used to assess tissue protein expression profiles.

### Definition of Protein–Protein Interaction (PPI) Modules

Genes form PPI networks at any level that fulfill biological roles, and perturbing protein networks is an ordinary characteristic of disease-associated mutations. Construction of a PPI network was studied by STRING tool (https:// www.string-db.org/) (an online database for the retrieval of interacting genes) (Szklarczyk et al., 2011). Gene ontology (GO) enrichment was performed for the categories gene ontology biological process, molecular function, cellular compartment, and Kyoto Encyclopedia of Genes and Genomes (KEGG) (https://www.kegg.jp/) (Yu et al., 2012).

### Quantification of Immune Infiltration Based on Key Genes

Immune infiltrate estimation was explored to comprehensively analyze the correlation between *PLOD* genes and tumor-infiltrating immune components by the Tumor Immune Estimation Resource (TIMER) platform (http://timer.cistrome.org/) (Li et al., 2017). The infiltrating immune cells including B cells, CD4^+^ T cells, CD8^+^ T cells, neutrophils, macrophages, and dendritic cells.

### Tumor-Immune System Interaction Database (TISIDB) Analysis

The integrated repository portal for tumor-immune system interactions with *PLODs* genes was assessed in TISIDB (Ru et al., 2019). Based on the gene expression profile, the relative abundance of tumor-infiltrating lymphocytes (TILs) was inferred by Gene Set Variation Analysis (GSVA). Spearman’s correlation test was conducted to explore the relation between numerical variables.

### PrognoScan Database

PrognoScan database (http://dna00.bio.kyutech.ac.jp/PrognoScan/index.html) (Mizuno et al., 2009) provides a powerful online platform for evaluating potential tumor markers and therapeutic targets, as well as a tool for assessing the biological association between gene expression and prognosis. The correlation between *PLOD* gene expression and survival in LUAD was analyzed by the PrognoScan database (jacob-00182-CANDF, jacob-00182-MSK, jacob-00182-UM GSE31210, HARVARD-LC, MICHIGAN-LC, and jacob-00182-CANDF).

### Prognostic Value of *PLODs*

The Gene Expression Profiling Interactive Analysis (GEPIA) contains high-throughput RNA sequencing data (TCGA and GTEx databases). Prognostic value, including OS and RFS, based on *PLOD* expression was generated by GEPIA database (http://gepia2.cancer-pku.cn/) (Tang et al., 2017).

### Statistical Analyses

Data were performed with R (V 3.5.3) and graphs were generated using the ggplot2 package in R. Statistical significance was measured by paired *t*-test and Mann–Whitney *U* test. The “pROC” package (Robin et al., 2011) was performed to calculate the cut-off value. Kaplan–Meier curves were determined by using log-rank tests.

## Results

### Pan-Cancer Analyses

**FIGURE 1:**
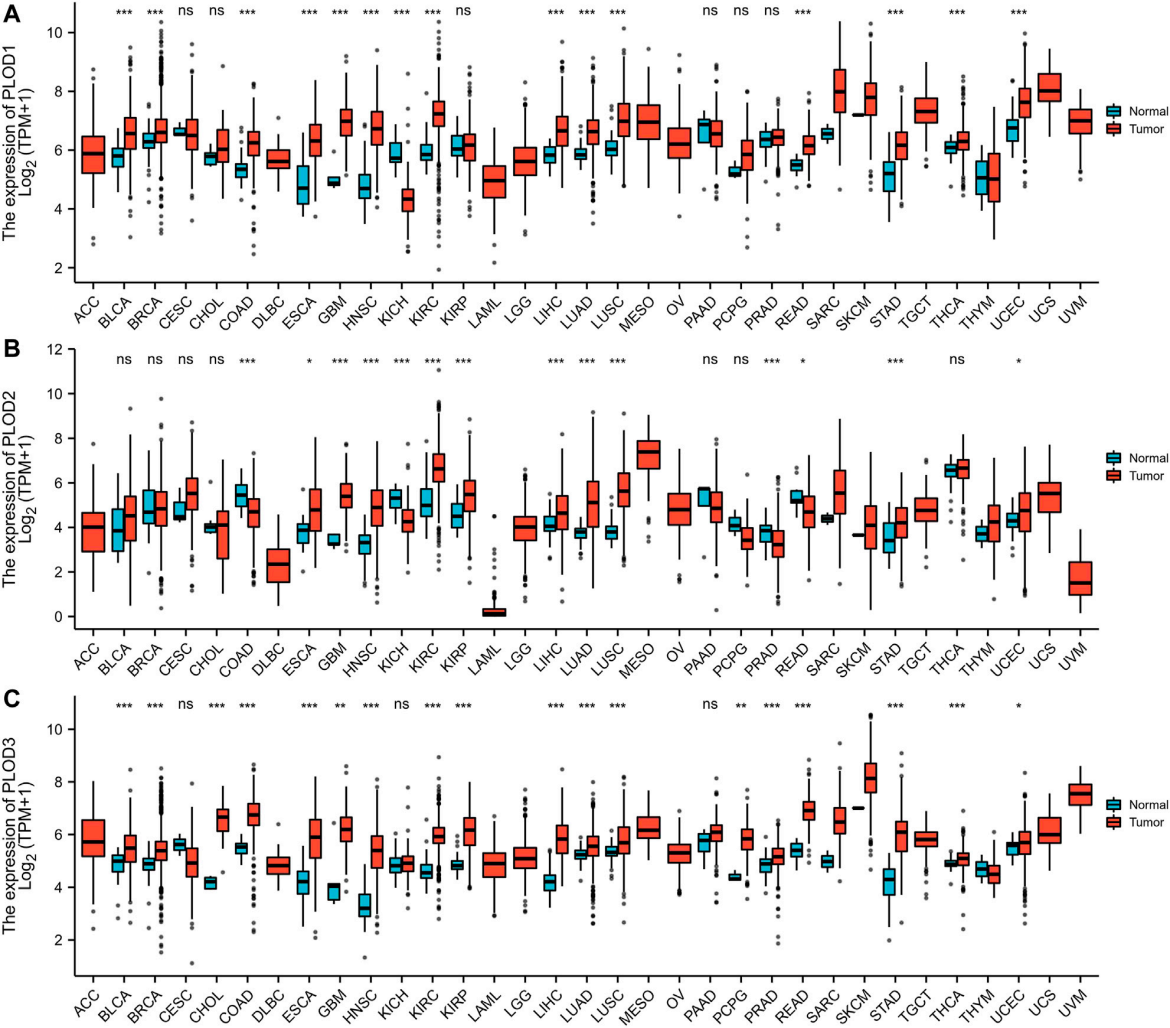
Pan-cancer analyses. **(A)** mRNA expression of PLOD1 upregulated in 14 of 33 cancer types; **(B)** mRNA expression of PLOD2 upregulated in 11 of 33 cancer types; **(C)** mRNA expression of PLOD3 upregulated in 17 of 33 cancer types. *p < 0.05, *p < 0.01, ***p < 0.001. ns, no significance; ACC, adrenocortical carcinoma; BLCA, bladder urothelial carcinoma; BRCA, breast invasive carcinoma; CESC, cervical squamous cell carcinoma and endocervical adenocarcinoma; CHOL, cholangiocarcinoma; COAD, colon adenocarcinoma; DLBC, lymphoid neoplasm diffuse large B-cell lymphoma; ESCA, esophageal carcinoma; GBM, glioblastoma multiforme; HNSC, head and neck squamous cell carcinoma; KICH, kidney chromophobe; KIRC, kidney renal clear cell carcinoma; KIRP, kidney renal papillary cell carcinoma; LAML, acute myeloid leukemia; LGG, brain lower grade glioma; LIHC, liver hepatocellular carcinoma; LUAD, lung adenocarcinoma; LUSC, lung squamous cell carcinoma; MESO, mesothelioma; OV, ovarian serous cystadenocarcinoma; PRAD, prostate adenocarcinoma; PCPG, pheochromocytoma and paraganglioma; READ, rectum adenocarcinoma; SARC, sarcoma; SKCM, skin cutaneous melanoma; STAD, stomach adenocarcinoma; THCA, thyroid carcinoma; THYM, thymoma; UCEC, uterine corpus endometrial carcinoma; UCS, uterine carcinosarcoma; UVM, uveal melanoma.

The mRNA expression levels of *PLODs* were analyzed using TCGA database. Our analysis revealed that 14 of 33 cancer types exhibited the highest mRNA expression of *PLOD1* among the TCGA cohorts. Compared with normal tissues, PLOD2 was significantly upregulated in 11 cancer types. In addition, *PLOD3* expression was markedly increased in 17 cancer types. These data imply that *PLODs* are often overexpressed across different cancer types, especially LUAD (Figure 1). The detailed data are presented in Supplementary Tables 1–3.

### mRNA Expression

**FIGURE 2:**
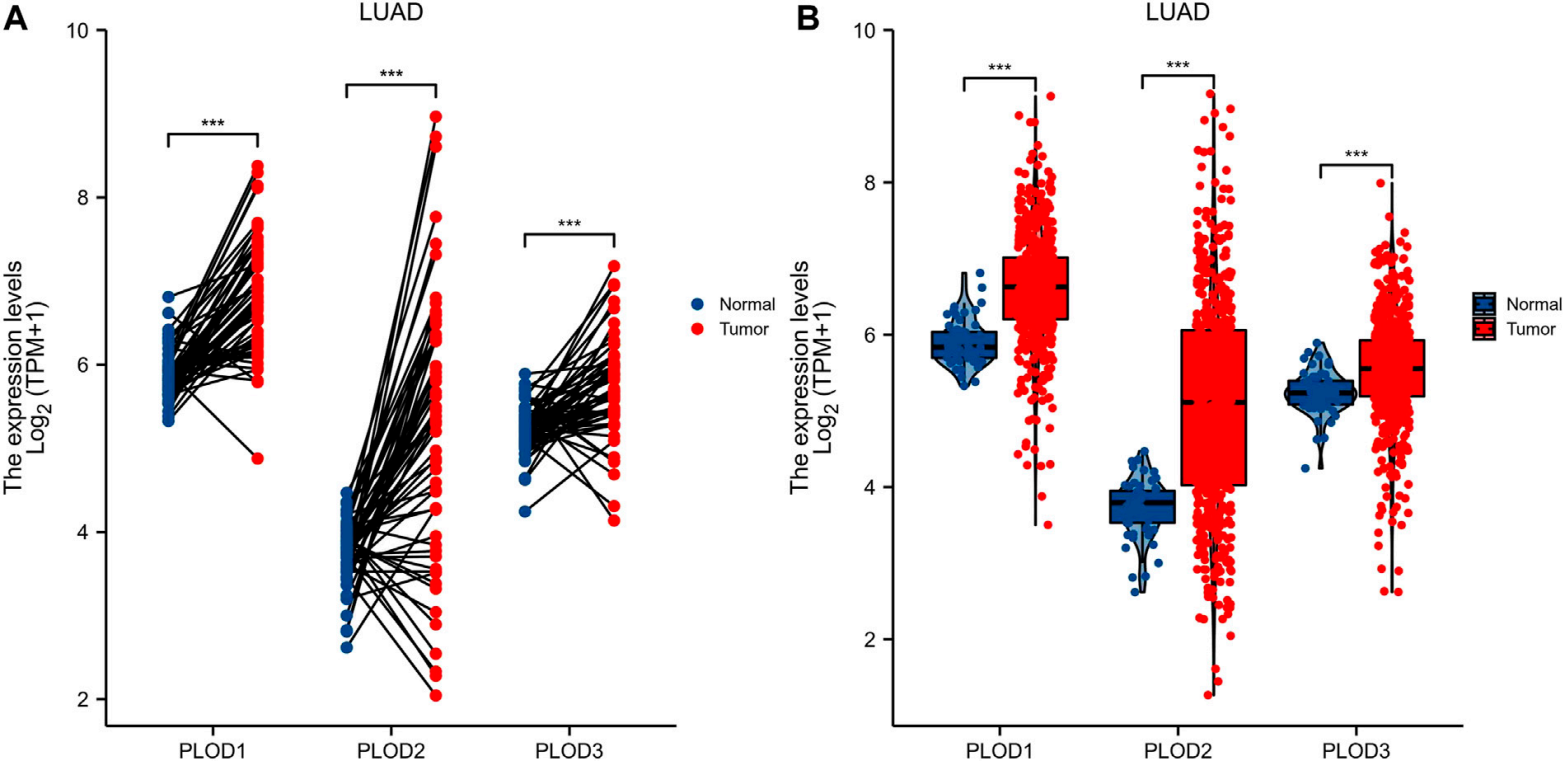
*PLOD* mRNA expression. Through TCGA database data screening and analysis, the expression of *PLOD* family genes in normal tissues and LUAD tissue samples was determined. **(A)** mRNA expression levels of *PLOD1*, *PLOD2*, and *PLOD3* in LUAD tissues and matched adjacent normal samples. **(B)** mRNA expression levels of *PLOD* family genes in 535 lung adenocarcinoma samples and 59 normal samples. The results showed that, compared with normal lung tissue, the expression of *PLOD1*, *PLOD2*, and *PLOD3* mRNA in LUAD increased significantly.

Expression of *PLODs* in normal lung (59 cases) and LUAD (535 cases) tissues from TCGA database were analyzed. Expression levels of *PLOD* mRNAs in LUAD tumor tissues were higher than those in adjacent normal tissues (Figure 2A). In subsequent analyses, the Mann–Whitney *U* test for unpaired data showed a significant difference between the expression levels of *PLOD* mRNAs in LUAD tissues and adjacent normal tissues (Figure 2B).

### Protein Pathological Expression in Lung Tissue

**FIGURE 3:**
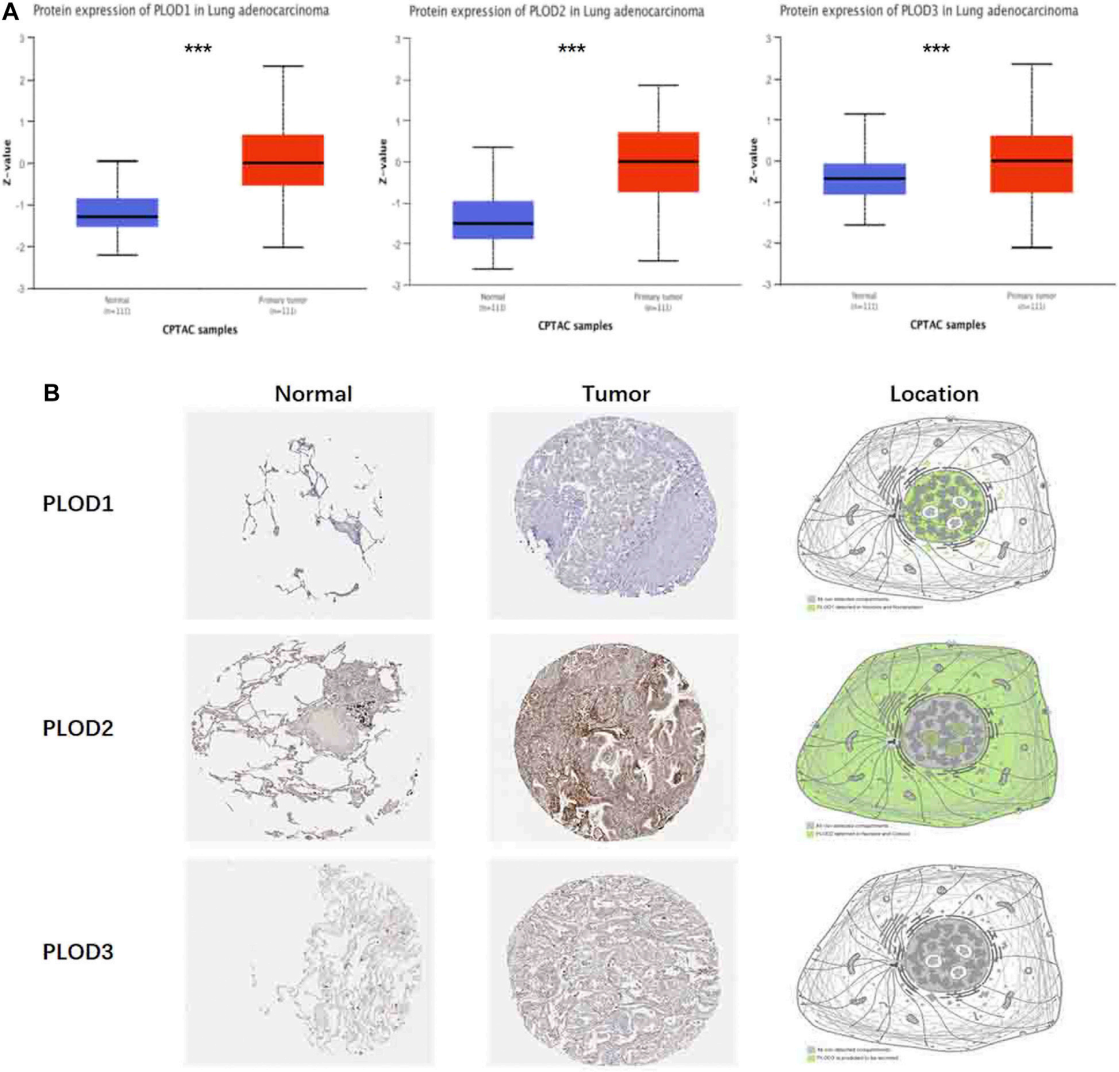
Protein pathological expression in lung tissue. To facilitate access to the protein expression of *PLOD1*, *PLOD2*, and *PLOD3* in LUAD, CPTAC data were integrated with the UALCAN data portal **(A)**, and the differential expression and location of *PLOD1-3* were observed in the HPA (***p < 0.001) **(B)**. *PLOD1*: normal tissue, https://www.proteinatlas.org/ENSG00000083444-*PLOD1*/tissue/lung; tumor tissue, https://www.proteinatlas.org/ENSG00000083444-*PLOD1*/pathology/lung + cancer. *PLOD2*: normal tissue, https://www.proteinatlas.org/ENSG00000152952-*PLOD2*/tissue/lung; tumor tissue, https://www.proteinatlas.org/ENSG00000152952-*PLOD2*/pathology. *PLOD3*: normal tissue, https://www.proteinatlas.org/ENSG00000106397-*PLOD3*/tissue/lung; tumor tissue, https://www.proteinatlas.org/ENSG00000106397-*PLOD3*/pathology/lung + cancer.

To determine PLOD protein expression, we mined these data using CPTAC with UALCAN. We found that the protein expression of *PLOD*s in LUAD was significantly higher (Figure 3A). Transcript data from the HPA and the corresponding staining patterns of PLODs were further supported by TCGA datasets. As shown in Figure 3B, PLOD1 is located in human cell vesicles and the nucleoplasm, PLOD2 is located in the nucleoli and cytosol, *and* PLOD3 is secreted by cells. In line with previous data, both mRNA and protein were overexpressed in LUAD tissues.

### Relationships Between *PLOD* Expression Data and LUAD Clinicopathological Characteristics

**FIGURE 4:**
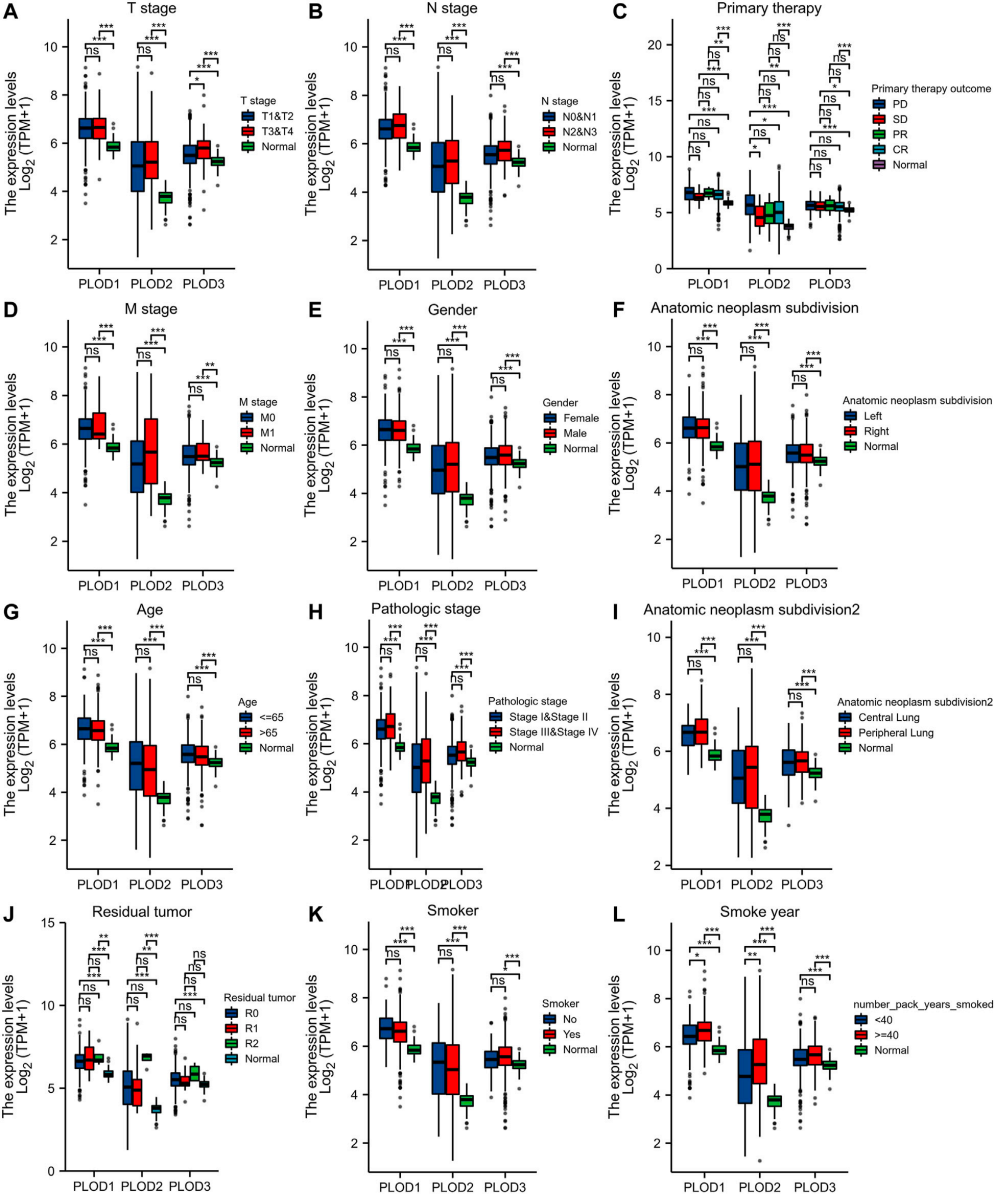
Evaluate relationship between mRNA expression level and various clinicopathological characteristics. The association of mRNA with clinical pathological characteristics including T stage **(A)**, lymph node metastases **(B)**, primary therapy outcome **(C)**, M stage **(D)**, sex **(E)**, anatomic neoplasm subdivision **(F)**, age **(G)**, pathologic stage **(H)**, anatomic neoplasm subdivision 2 **(I)**, residual tumor **(J)**, smoker **(K)**, and smoking status **(L)** are demonstrated in this plot. ns, no significance, *p < 0.05, **p < 0.01, ***p < 0.001.

We applied the Mann–Whitney *U* test and logistic regression analysis. As shown in Table 1, Table 2, Table 3, and Figure 4A-L, *PLOD* expression was correlated with clinical stage, T stage, OS, histopathological grade, and smoking history. However, *PLOD* expression and other clinicopathological characteristics, including age, smoking status, M stage, sex, and anatomical location (right and left; periphery and center), were not statistically significant. In summary, these results indicate that *PLOD1* and *PLOD2* may serve as biomarkers for poor prognosis of LUAD.

**TABLE 1:** Clinical Characteristics of the LUAD patients (TCGA)

Characteristic	Levels	Low expression of PLOD1	High expression of PLOD1	*p*-value
N		267	268	
T stage, *n* (%)	T1	107 (20.1)	68 (12.8)	0.003
	T2	126 (23.7)	163 (30.6)	
	T3	24 (4.5)	25 (4.7)	
	T4	8 (1.5)	11 (2.1)	
N stage, *n* (%)	N0	184 (35.5)	164 (31.6)	0.043
	N1	42 (8.1)	53 (10.2)	
	N2	29 (5.6)	45 (8.7)	
	N3	0 (0)	2 (0.4)	
M stage, *n* (%)	M0	174 (45.1)	187 (48.4)	0.186
	M1	16 (4.1)	9 (2.3)	
Pathologic stage, *n* (%)	Stage I	162 (30.7)	132 (25)	0.004
	Stage II	52 (9.9)	71 (13.5)	
	Stage III	32 (6.1)	52 (9.9)	
	Stage IV	17 (3.2)	9 (1.7)	
Primary therapy outcome, *n* (%)	PD	30 (6.7)	41 (9.2)	0.073
	SD	25 (5.6)	12 (2.7)	
	PR	2 (0.4)	4 (0.9)	
	CR	167 (37.4)	165 (37)	
Gender, *n* (%)	Female	140 (26.2)	146 (27.3)	0.699
	Male	127 (23.7)	122 (22.8)	
Race, *n* (%)	Asian	4 (0.9)	3 (0.6)	0.431
	Black or African American	32 (6.8)	23 (4.9)	
	White	201 (42.9)	205 (43.8)	
Age, *n* (%)	≤65	123 (23.8)	132 (25.6)	0.429
	>65	136 (26.4)	125 (24.2)	
Residual tumor, *n* (%)	R0	177 (47.6)	178 (47.8)	0.721
	R1	6 (1.6)	7 (1.9)	
	R2	1 (0.3)	3 (0.8)	
Anatomic neoplasm subdivision, *n* (%)	Left	104 (20)	101 (19.4)	0.913
	Right	157 (30.2)	158 (30.4)	
Anatomic neoplasm subdivision2, *n* (%)	Central lung	30 (15.9)	32 (16.9)	1.000
	Peripheral lung	60 (31.7)	67 (35.4)	
number_pack_years_smoked, *n* (%)	<40	114 (30.9)	74 (20.1)	<0.001
	≥40	77 (20.9)	104 (28.2)	
Smoker, *n* (%)	No	34 (6.5)	41 (7.9)	0.465
	Yes	226 (43.4)	220 (42.2)	
OS event, *n* (%)	Alive	183 (34.2)	160 (29.9)	0.041
	Dead	84 (15.7)	108 (20.2)	
DSS event, *n* (%)	Alive	195 (39.1)	184 (36.9)	0.148
	Dead	52 (10.4)	68 (13.6)	
PFI event, *n* (%)	Alive	158 (29.5)	151 (28.2)	0.565
	Dead	109 (20.4)	117 (21.9)	
Age, median (IQR)		67 (59, 72)	65 (58, 72)	0.665
number_pack_years_smoked, median (IQR)		30 (20, 50)	40 (25, 57.5)	0.002

**TABLE 2:** Clinical characteristics of the LUAD patients (TCGA)

Characteristic	Levels	Low expression of PLOD2	High expression of PLOD2	*p*-value
N		267	268	
T stage, *n* (%)	T1	102 (19.2)	73 (13.7)	0.049
	T2	135 (25.4)	154 (28.9)	
	T3	22 (4.1)	27 (5.1)	
	T4	7 (1.3)	12 (2.3)	
N stage, *n* (%)	N0	181 (34.9)	167 (32.2)	0.238
	N1	42 (8.1)	53 (10.2)	
	N2	34 (6.6)	40 (7.7)	
	N3	0 (0)	2 (0.4)	
M stage, *n* (%)	M0	176 (45.6)	185 (47.9)	0.304
	M1	9 (2.3)	16 (4.1)	
Pathologic stage, *n* (%)	Stage I	162 (30.7)	132 (25)	0.044
	Stage II	54 (10.2)	69 (13.1)	
	Stage III	38 (7.2)	46 (8.7)	
	Stage IV	9 (1.7)	17 (3.2)	
Primary therapy outcome, *n* (%)	PD	25 (5.6)	46 (10.3)	0.029
	SD	23 (5.2)	14 (3.1)	
	PR	3 (0.7)	3 (0.7)	
	CR	171 (38.3)	161 (36.1)	
Gender, *n* (%)	Female	152 (28.4)	134 (25)	0.129
	Male	115 (21.5)	134 (25)	
Race, *n* (%)	Asian	3 (0.6)	4 (0.9)	0.114
	Black or African American	35 (7.5)	20 (4.3)	
	White	200 (42.7)	206 (44)	
Age, *n* (%)	≤65	119 (23.1)	136 (26.4)	0.113
	>65	141 (27.3)	120 (23.3)	
Residual tumor, *n* (%)	R0	178 (47.8)	177 (47.6)	0.122
	R1	8 (2.2)	5 (1.3)	
	R2	0 (0)	4 (1.1)	
Anatomic neoplasm subdivision, *n* (%)	Left	106 (20.4)	99 (19)	0.691
	Right	156 (30)	159 (30.6)	
Anatomic neoplasm subdivision2, *n* (%)	Central lung	32 (16.9)	30 (15.9)	0.357
	Peripheral lung	55 (29.1)	72 (38.1)	
number_pack_years_smoked, *n* (%)	<40	110 (29.8)	78 (21.1)	0.006
	≥40	79 (21.4)	102 (27.6)	
Smoker, *n* (%)	No	33 (6.3)	42 (8.1)	0.327
	Yes	227 (43.6)	219 (42)	
OS event, *n* (%)	Alive	183 (34.2)	160 (29.9)	0.041
	Dead	84 (15.7)	108 (20.2)	
DSS event, *n* (%)	Alive	199 (39.9)	180 (36.1)	0.034
	Dead	49 (9.8)	71 (14.2)	
PFI event, *n* (%)	Alive	165 (30.8)	144 (26.9)	0.072
	Dead	102 (19.1)	124 (23.2)	
Age, median (IQR)		67 (59, 72)	65 (58, 72)	0.267
number_pack_years_smoked, meidan (IQR)		33 (20, 50)	40 (25, 56)	0.004

**TABLE 3:** Clinical characteristics of the LUAD patients (TCGA)

Characteristic	Levels	Low expression of PLOD3	High expression of PLOD3	*p*-value
N		267	268	
T stage, *n* (%)	T1	103 (19.4)	72 (13.5)	0.007
	T2	138 (25.9)	151 (28.4)	
	T3	16 (3)	33 (6.2)	
	T4	9 (1.7)	10 (1.9)	
N stage, *n* (%)	N0	178 (34.3)	170 (32.8)	0.371
	N1	44 (8.5)	51 (9.8)	
	N2	33 (6.4)	41 (7.9)	
	N3	0 (0)	2 (0.4)	
M stage, *n* (%)	M0	188 (48.7)	173 (44.8)	1.000
	M1	13 (3.4)	12 (3.1)	
Pathologic stage, *n* (%)	Stage I	162 (30.7)	132 (25)	0.036
	Stage II	50 (9.5)	73 (13.9)	
	Stage III	37 (7)	47 (8.9)	
	Stage IV	13 (2.5)	13 (2.5)	
Primary therapy outcome, *n* (%)	PD	30 (6.7)	41 (9.2)	0.648
	SD	19 (4.3)	18 (4)	
	PR	3 (0.7)	3 (0.7)	
	CR	167 (37.4)	165 (37)	
Gender, *n* (%)	Female	148 (27.7)	138 (25.8)	0.409
	Male	119 (22.2)	130 (24.3)	
Race, *n* (%)	Asian	3 (0.6)	4 (0.9)	0.876
	Black or African American	29 (6.2)	26 (5.6)	
	White	203 (43.4)	203 (43.4)	
Age, *n* (%)	≤65	120 (23.3)	135 (26.2)	0.159
	>65	140 (27.1)	121 (23.4)	
Residual tumor, *n* (%)	R0	183 (49.2)	172 (46.2)	0.424
	R1	9 (2.4)	4 (1.1)	
	R2	2 (0.5)	2 (0.5)	
Anatomic neoplasm subdivision, *n* (%)	Left	99 (19)	106 (20.4)	0.542
	Right	162 (31.2)	153 (29.4)	
Anatomic neoplasm subdivision2, *n* (%)	Central lung	28 (14.8)	34 (18)	0.932
	Peripheral lung	55 (29.1)	72 (38.1)	
number_pack_years_smoked, *n* (%)	<40	100 (27.1)	88 (23.8)	0.052
	≥40	77 (20.9)	104 (28.2)	
Smoker, *n* (%)	No	42 (8.1)	33 (6.3)	0.309
	Yes	218 (41.8)	228 (43.8)	
OS event, *n* (%)	Alive	181 (33.8)	162 (30.3)	0.093
	Dead	86 (16.1)	106 (19.8)	
DSS event, *n* (%)	Alive	193 (38.7)	186 (37.3)	0.386
	Dead	55 (11)	65 (13)	
PFI event, *n* (%)	Alive	158 (29.5)	151 (28.2)	0.565
	Dead	109 (20.4)	117 (21.9)	
Age, median (IQR)		67 (60, 72.25)	65 (58, 72)	0.135
number_pack_years_smoked, meidan (IQR)		35 (20, 50)	40 (25, 53.25)	0.140

### Potential Biomarkers for Distinguishing LUAD Samples From Normal Samples

**FIGURE 5:**
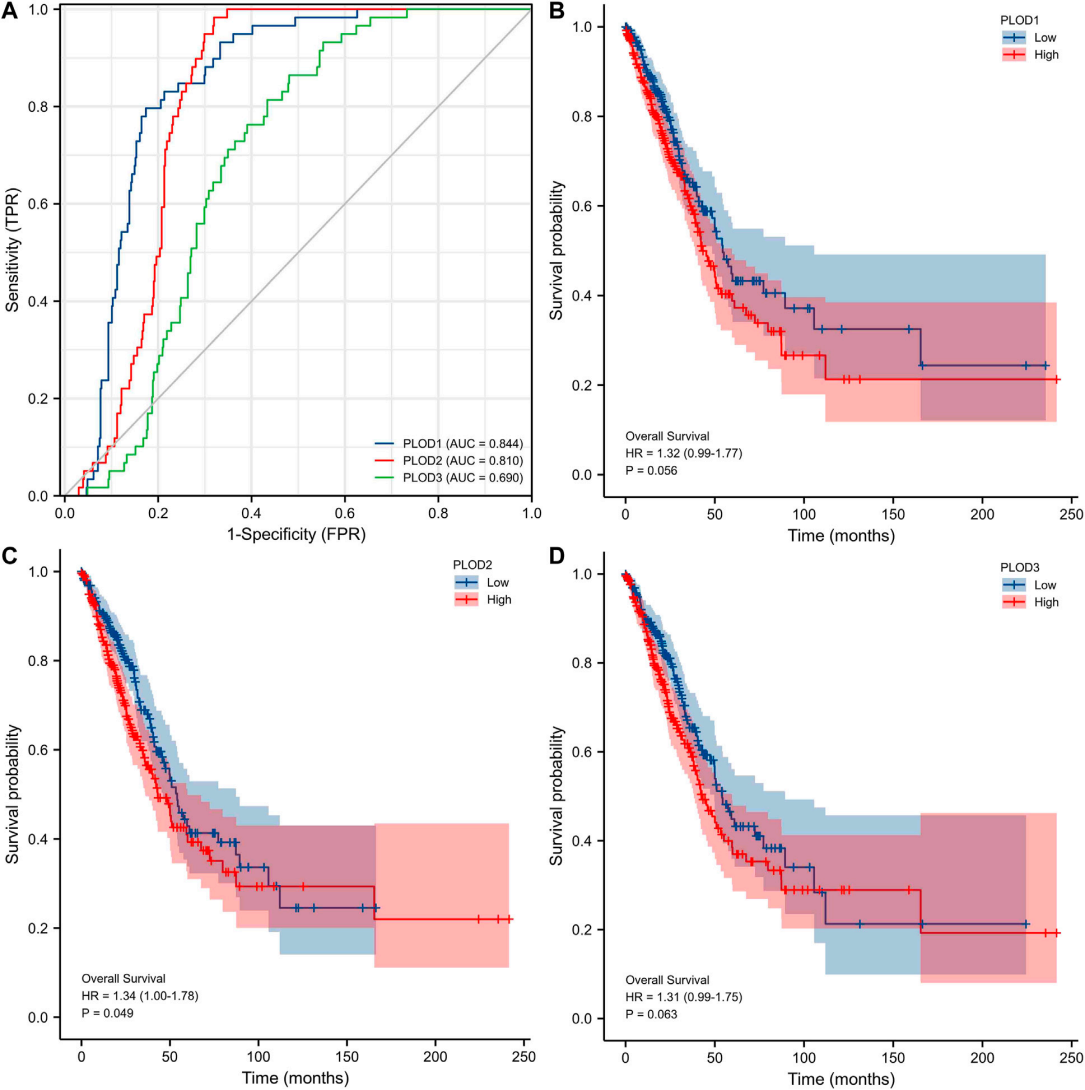
Prospective biomarkers for distinguishing LUAD from normal samples. The survival ROC package in the R language was used to generate the ROC curve. **(A)** ROC curve showing that *PLOD1* and *PLOD2* can distinguish lung adenocarcinoma tissue from healthy controls. **(B**–**D)** The Kaplan–Meier curve of the *PLOD* gene family showed that the OS of LUAD patients with high *PLOD2* mRNA expression was shorter than that of patients with low *PLOD2*, and the difference was statistically significant.

ROC curve analysis showed (Figure 5A) significant predictive potential for *PLOD1* (AUC = 0.844, CI = 0.806–0.881) and *PLOD2* (AUC = 0.810, CI = 0.776–0.844). However, the predictive potential for *PLOD3* was lower (AUC = 0.690, CI = 0.641–0.740). DeLong’s test showed that *PLOD1* was superior to *PLOD2* in predicting tumor and normal outcomes, but the test was not statistically significant (*p* = 0.148); *PLOD1* was significantly superior to *PLOD3* (*p* ≤ 0.01). The diagnostic efficiency of *PLOD2* was significantly better than that of *PLOD3* (*p* ≤ 0.01). Kaplan–Meier curves for the three *PLOD* family members showed that the OS of LUAD patients with high *PLOD*2 mRNA expression was shorter than that of patients with low *PLOD2*, and the difference was statistically significant.

### PPI Network Analysis and Biological Function Annotation

**FIGURE 6:**
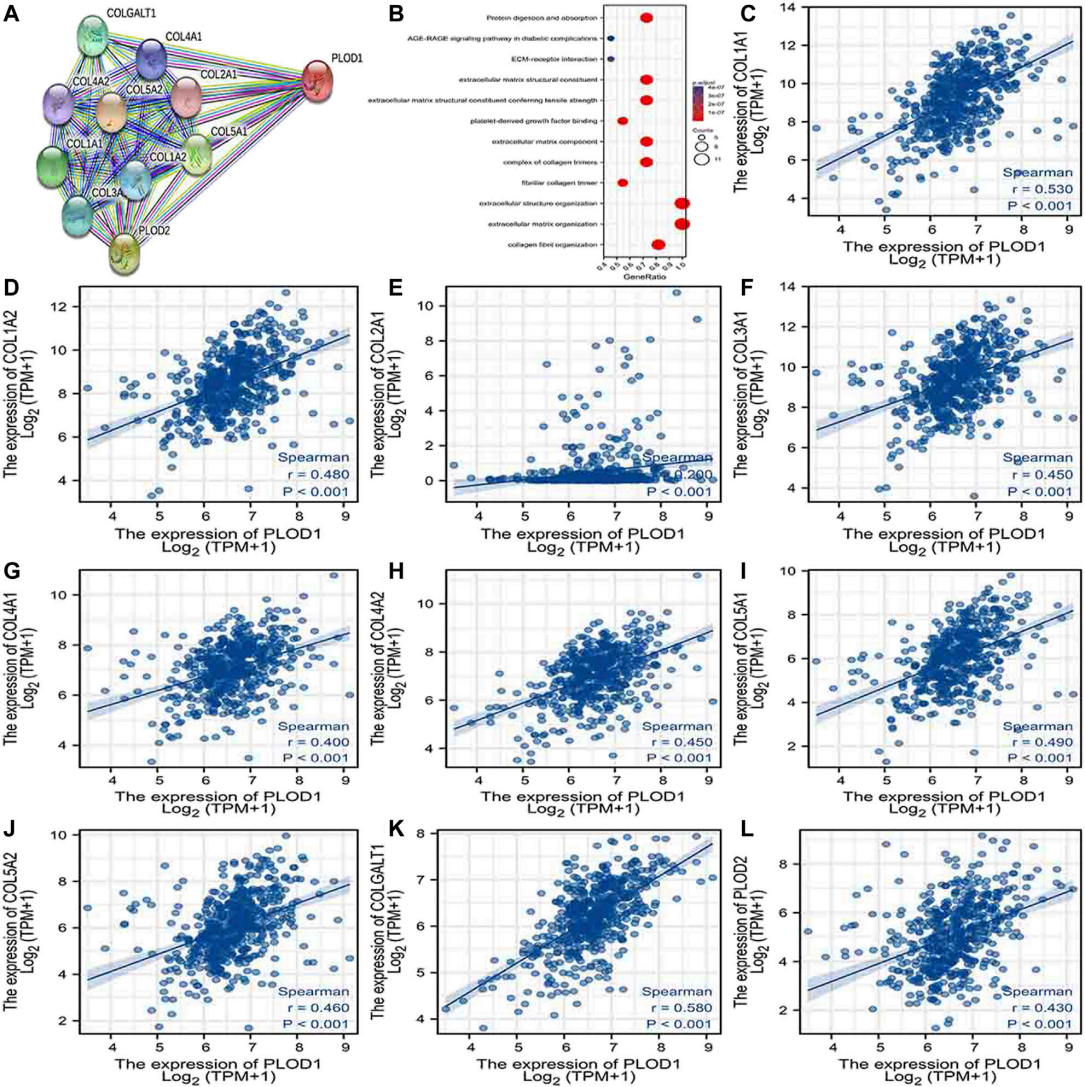
*PLOD1* PPI network and biological function annotation. **(A)** Network of PLOD1 and its co-expressed genes. **(B)** Functional enrichment analysis of 10 network genes. PLOD1 is associated with collagen fibril organization, extracellular matrix organization, and extracellular structural organization. These genes are involved in protein digestion and absorption, ECM–receptor interactions, and the AGE-RAGE signaling pathway in diabetic complications. **(C–L)** Correlations between the expression of PLOD1 and co-expressed genes in LUAD. COL1A1, collagen alpha-1(I) chain; COL1A2, collagen alpha-2(I) chain; COL2A1, collagen alpha-1(II) chain; COL3A1, collagen alpha-1(III) chain; COL4A1, collagen alpha-1(IV) chain; COL4A2, collagen alpha-2(IV) chain; COL5A1, collagen alpha-1(V) chain; COL5A2, collagen alpha-2(V) chain; COLGALT1, procollagen galactosyltransferase 1; *PLOD2*: procollagen-lysine,2-oxoglutarate 5-dioxygenase 2.

**FIGURE 7:**
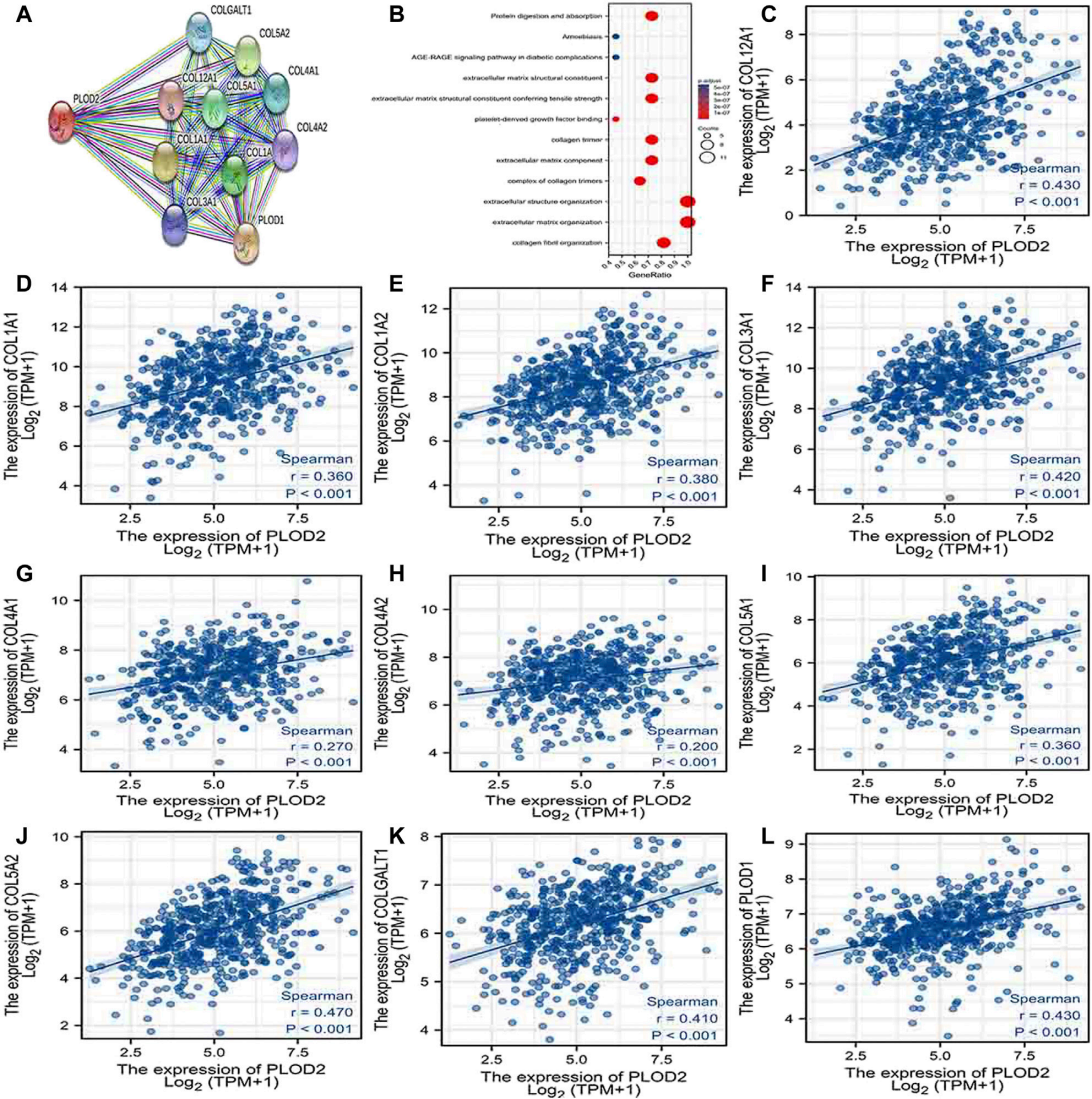
*PLOD2* PPI network and biological function annotation. **(A)** Network of PLOD2 and its co-expressed genes. **(B)** Functional enrichment analysis of 10 network genes. PLOD2 was associated with collagen fibril organization, extracellular matrix organization, and extracellular structure organization. These genes are involved in protein digestion and absorption, amoebiasis, and AGE-RAGE signaling in diabetes. **(C–L)** Correlations between the expression of PLOD1 and co-expressed genes in lung adenocarcinoma. COL12A1, collagen alpha-1 (XII) chain; COL1A1, collagen alpha-1(I) chain; COL1A2, collagen alpha-2(I) chain; COL3A1, collagen alpha-1(III) chain; COL4A1, collagen alpha-1(IV) chain; COL4A2, collagen alpha-2(IV) chain; COL5A1, collagen alpha-1(V) chain; COL5A2, collagen alpha-2(V) chain; COLGALT1, procollagen galactosyltransferase 1; *PLOD1*: procollagen-lysine,2-oxoglutarate 5-dioxygenase 1.

**FIGURE 8:**
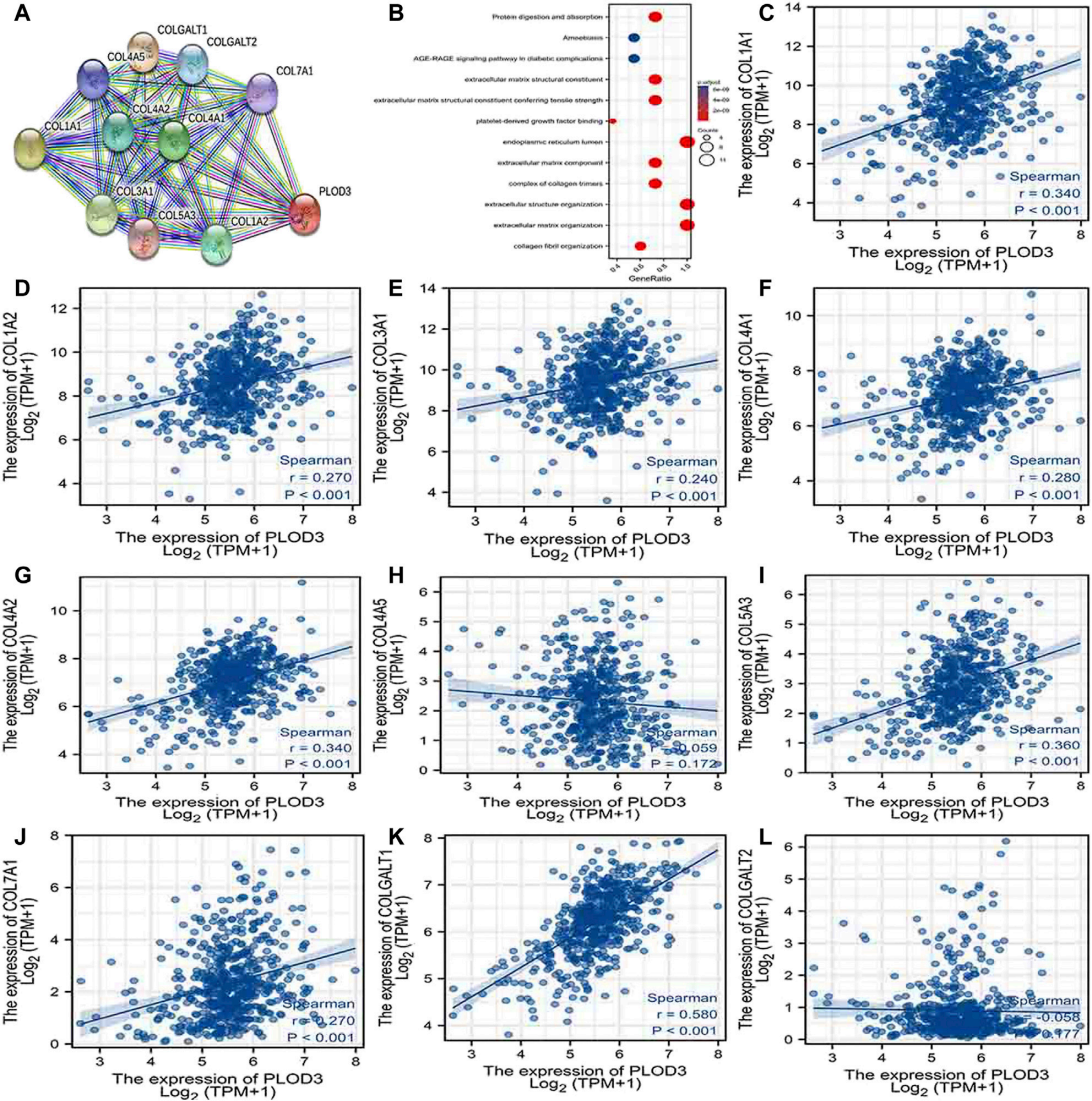
*PLOD3* PPI network and biological function annotation. **(A)** Network of PLOD3 and its co-expressed genes. **(B)** Functional enrichment analysis of 10 network genes. PLOD3 was associated with collagen fibril organization, extracellular matrix organization, and extracellular structure organization. These genes were involved in protein digestion and absorption, amoebiasis, and AGE-RAGE signaling in diabetes. **(C–L)** Correlations between the expression of PLOD3 and co-expressed genes in lung adenocarcinoma. COL1A1, collagen alpha-1(I) chain; COL1A2, collagen alpha-2(I) chain; COL3A1, collagen alpha-1(III) chain; COL4A1, collagen alpha-1(IV) chain; COL4A2, collagen alpha-2(IV) chain; COL4A5, collagen alpha-5(IV) chain; COL5A3, collagen alpha-3(V) chain; COL7A1, collagen alpha-1(VII) chain; COLGALT1, procollagen galactosyltransferase 1; COLGALT2, procollagen galactosyltransferase 2.

Using the STRING database, Figure 6A, Figure 7A, and Figure 8A show networks of 10 co-expressed genes related to *PLODs* in LUAD patients. For *PLOD1*, the network included *COL1A1*, *COL1A2*, *COL2A1*, *COL3A1*, *COL4A1*, *COL4A2*, *COL5A1*, *COL5A2*, *COLGALT1*, and *PLOD2*. For *PLOD2*, the network included *COL12A1*, *COL1A1*, *COL1A2*, *COL3A1*, *COL4A1*, *COL4A2*, *COL5A1*, *COL5A2*, *COLGALT1*, and *PLOD1*. For *PLOD3*, the network included *COL1A1*, *COL1A2*, *COL3A1*, *COL4A1*, *COL4A2*, *COL4A5*, *COL5A3*, *COL7A1*, *COLGALT1*, and *COLGALT2*). As shown in Figure 6, Figure 7, and Figure 8B, the biological processes for *PLODs* were all related to collagen fibril, extracellular matrix, and extracellular structure organization. The functional annotations for *PLOD*1 were protein digestion and absorption, ECM–receptor interaction, and the AGE-RAGE signaling pathway in diabetic complications. The functional annotations of *PLOD*2 and *PLOD*3 were protein digestion and absorption, amoebiasis, and AGE-RAGE signaling pathways in diabetic complications. Correlation analyses between the expression of *PLODs* and TCGA LUAD co-expressed genes are shown in Figures 6C–L, Figures 7C–L, and Figures 8C–L.

### Associations With Target Genes and Immune Infiltration

**FIGURE 9:**
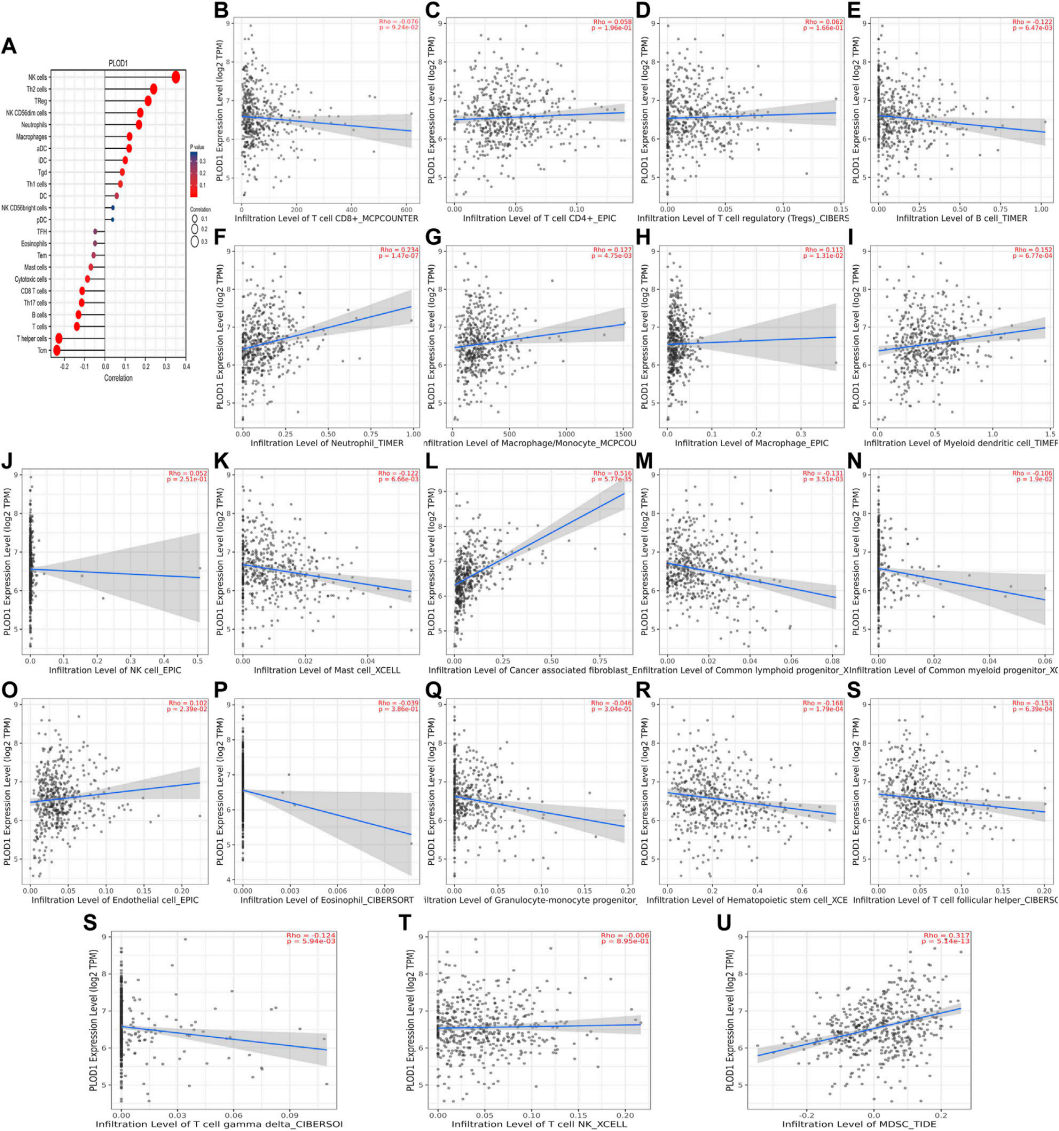
Association between *PLOD1* and immune infiltration. **(A)** Summary plot of the relationship between *PLOD1* expression and immune infiltration. **(B–U)** *PLOD1* expression is negatively related to tumor purity and has correlations with TFH, CD8^+^ T, TH, cytotoxic, mast, and B cells; on the contrary, it is positively correlated with Tregs, macrophages, and cancer-associated fibroblasts.

**FIGURE 10:**
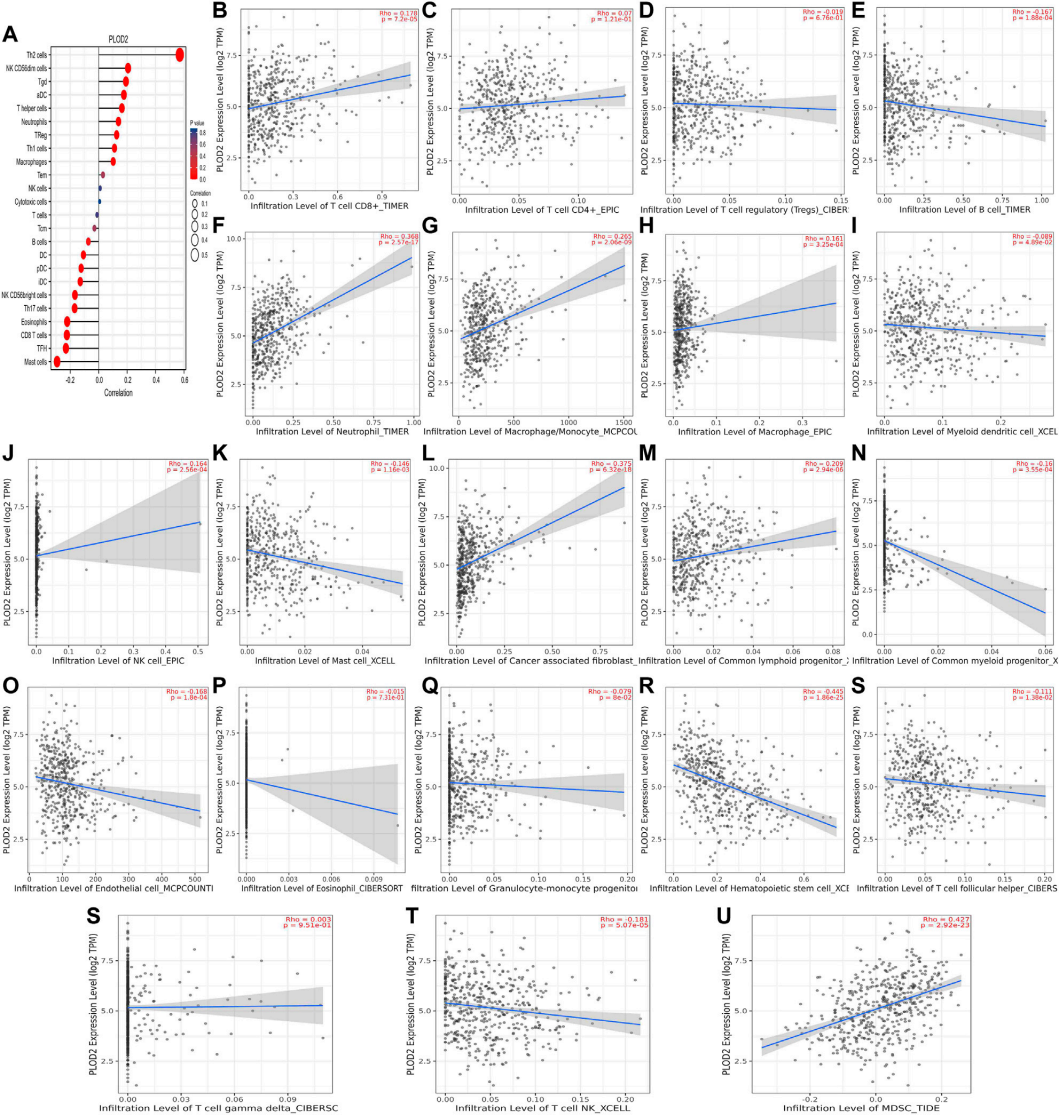
Association between *PLOD2* and immune infiltration. **(A)** Summary plot of the relationship between *PLOD2* expression and immune infiltration. **(B–U)** *PLOD2* expression is negatively related to tumor purity and has correlations with TFH, CD8^+^ T, dendritic, CD56^+^ NK, mast, and B cells; on the contrary, it is positively correlated with Tregs, macrophages, and cancer-associated fibroblasts.

**FIGURE 11:**
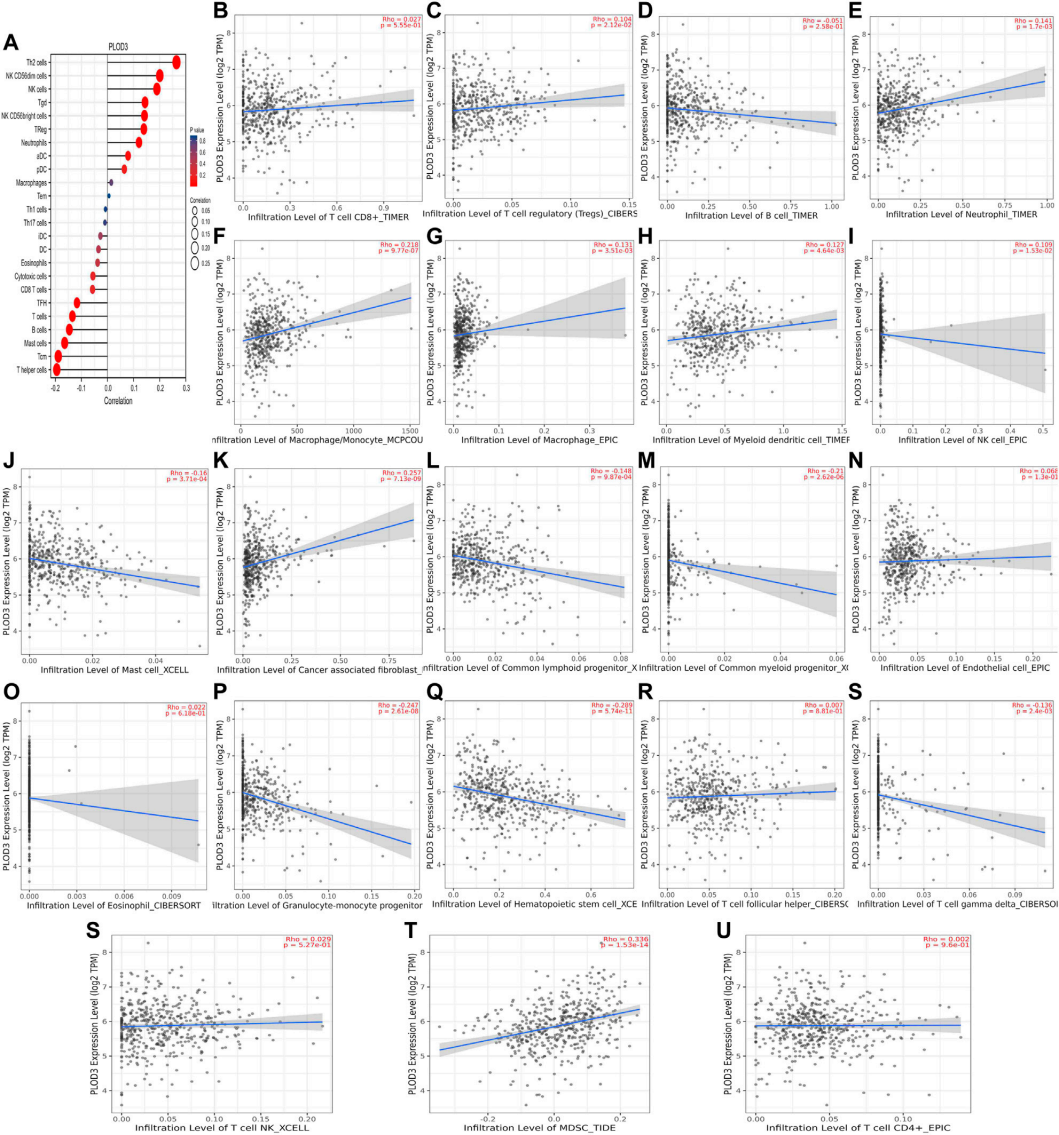
Association between *PLOD3* and immune infiltration. **(A)** Summary plot of the relationship between *PLOD2* expression and immune infiltration. **(B–U)** *PLOD3* expression is negatively related to tumor purity and has correlations with TFH, CD8^+^ T, TH, dendritic, cytotoxic, and mast and B cells; on the contrary, it is positively correlated with Tregs, macrophages, and cancer-associated fibroblast cells.

We used TCGA to analyze *PLODs* and overall immune components in tumor immunosuppressive networks (Figures 9A, 10, and 11A). We next expanded our analysis by interrogating TIMER (Figure 9B–L, Figure 10B–L, and Figure 11B–L). We found that in the LUAD patient cohort, high *PLOD* expression levels were negatively correlated with TFH cells, CD8 T cells, Tcm cells, and B-cell immune infiltration. In contrast, high *PLOD1-3* expression was positively correlated with Tregs, macrophages, and cancer-associated fibroblasts.

### Correlation of *PLOD* Expression With TILs

**FIGURE 12:**
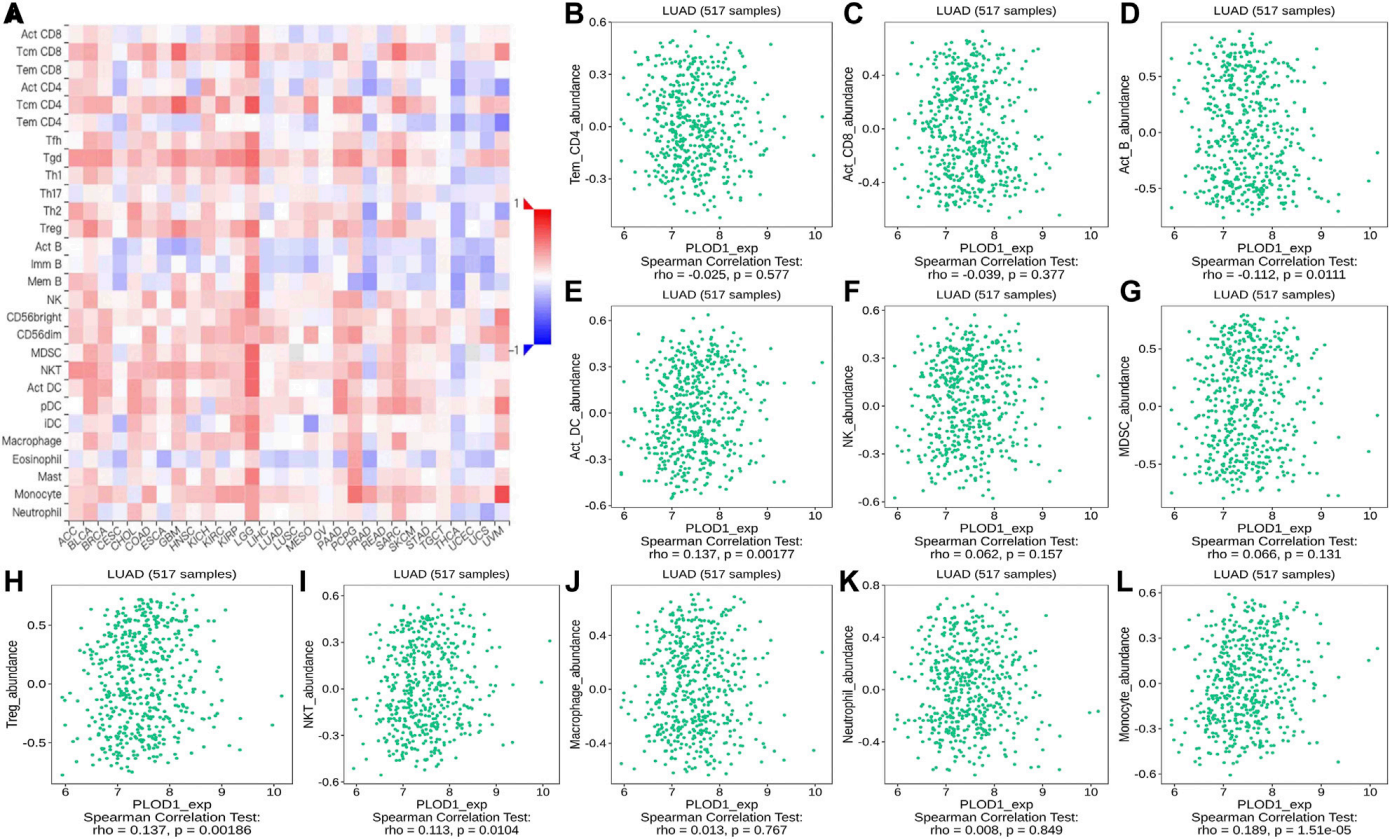
Correlation of *PLOD1* expression with TILs. **(A)** Heatmap of *PLOD1* expression in pan-cancer from the TISIDB database. **(B–L)** *PLOD1* expression level is significantly correlated with infiltrating levels of activated CD8^+^ T cells, activated CD4^+^ T cells, activated B cells, Treg cells, NKT cells, and macrophages.

**FIGURE 13:**
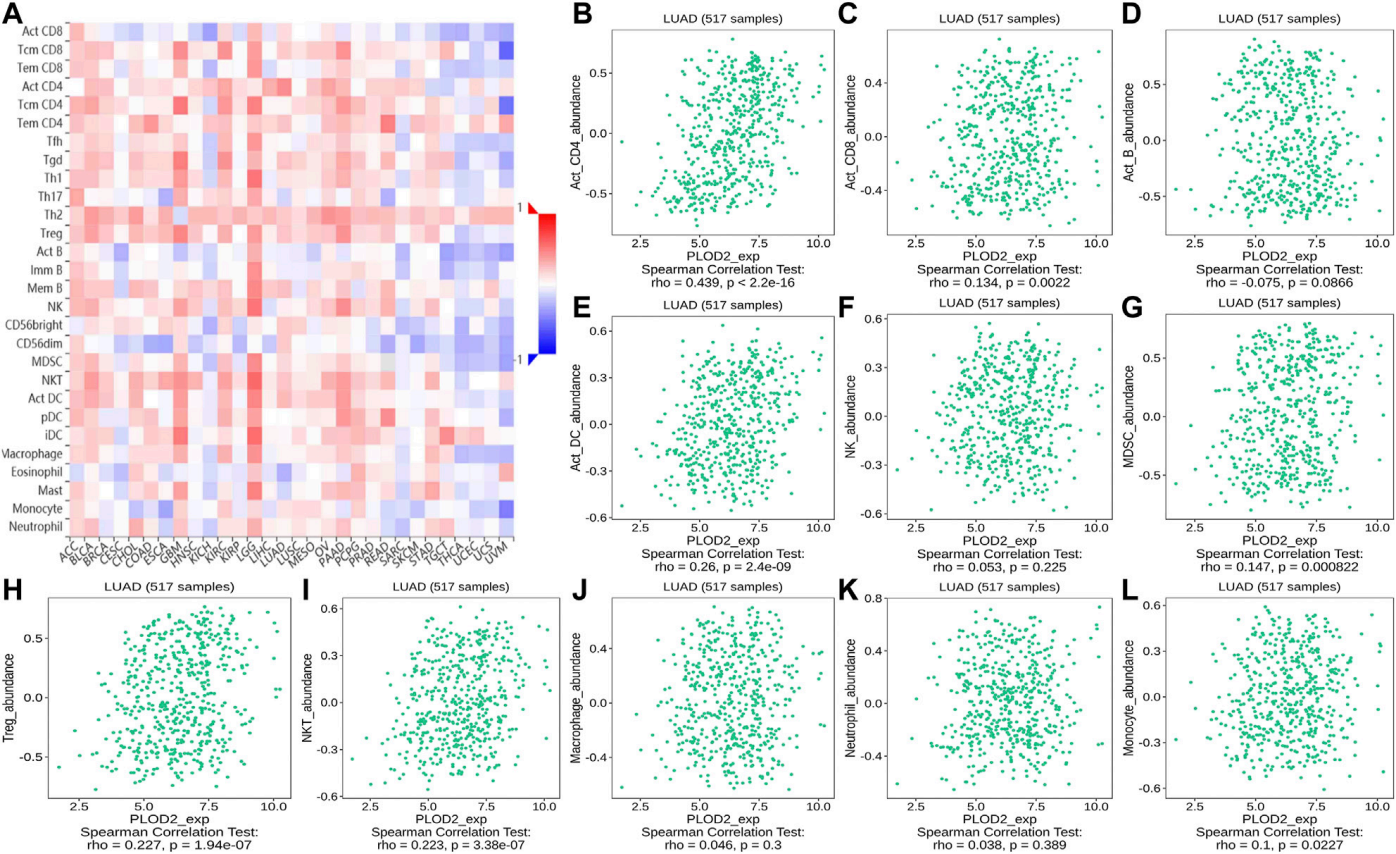
Correlation of *PLOD2* expression with TILs. **(A)** Heatmap of *PLOD2* expression in pan-cancer from the TISIDB database. **(B–L)** *PLOD2* expression level is significantly correlated with infiltrating levels of activated CD8^+^ T cells, activated CD4^+^ T cells, activated DCs, activated CD8^+^ T cells, MDSCs, Treg cells, and NKT cells.

**FIGURE 14:**
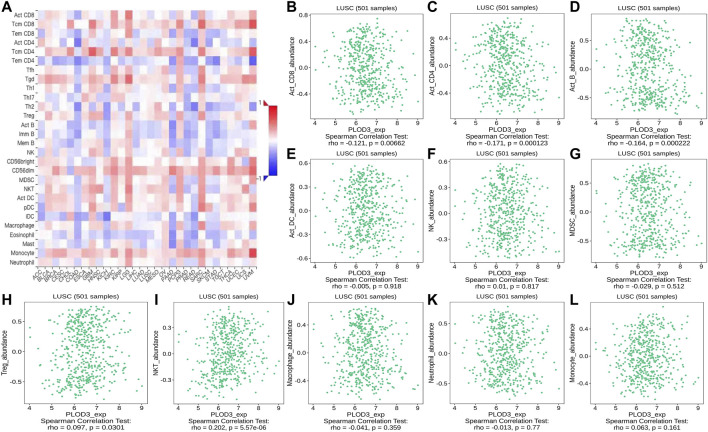
Correlation of *PLOD3* expression with TILs. **(A)** Heatmap of *PLOD3* expression in pan-cancer from the TISIDB database. **(B–L)** *PLOD3* expression level is significantly correlated with infiltrating levels of activated CD8^+^ T cells, activated CD4^+^ T cells, activated B cells, Treg cells, and NKT cells.

Using the TISIDB database, we found that the ratios of TIL subpopulations were highly correlated with *PLOD* expression in pan-cancer (Figure 12A, Figure 13A, and Figure 14A); representative pictures are shown in Figures 12B–L, Figures 13B–L, and Figures 14B–L. In general, the TISIDB results were similar to those obtained using TIMER.

## Discussion

Lung adenocarcinoma has high mortality, ranking first and second in cancer-type incidence in men and women in China, respectively. It is also the leading cause of cancer-related deaths worldwide. Owing to the combined effects of tobacco, aging, and air pollution, the incidence of lung cancer is rapidly increasing. The most common subtype of lung cancer is LUAD. Since it is typically diagnosed late in its progression, the 5-year OS is only 15%. Therefore, it is important to identify differentially expressed molecules to use as markers for its diagnosis (Li et al., 2016; Tang et al., 2020).

In the present study, *PLODs* were found to be overexpressed in LUAD tissues; therefore, they may be independent prognostic biomarkers. The level of *PLOD* mRNA expression was positively correlated with tumor progression. ROC analysis demonstrated that *PLODs* may be a promising diagnostic indicator for distinguishing LUAD from normal tissues. From Kaplan–Meier curves, we confirmed that high expression of *PLODs* was associated with a shorter survival. Thus, *PLODs* represent attractive potential prognostic biomarkers for LUAD. Furthermore, PLODs may play specific roles in immune evasion.

Lysine hydroxylase (LH) is encoded by three lysine hydroxylase genes (procollagen-lysine, 2-oxoglutarate 5-dioxygenase *PLOD1*, *PLOD2*, and *PLOD3*) and is a key enzyme that mediates collagen association. *PLOD* family genes belong to the 2-ketoglutarate-dependent dioxygenase family (Zhao et al., 2021). They form homodimers in the presence of Fe^2+^ and 2-ketoglutaric acid (2-OG) to hydroxylate single-chain procollagen lysines. Collagen that undergoes lysine hydroxylation is secreted by the cell. It can be crosslinked to form stable hydroxylysine pyridine chains (Eisinger-Mathason et al., 2013). When *PLOD1* and *PLOD2* are significantly overexpressed, hydroxylysine pyridine chains are generated (Heikkinen et al., 1994). This leads to excessive deposition of collagen fibers, which destroys the ECM structure. Eventually, overexpression of *PLODs* promotes tumor progression and metastasis.

PLOD1 catalyzes hydroxylation of lysyl residues on collagen type V. It was originally used as a biochemical and histochemical marker for the assessment of bone mineral density (Shin et al., 2020). With the development of genome and transcriptome sequencing, *PLOD1* mutations have been found to be associated with a variety of cancers (Xu et al., 2019). Wang et al. reported that *PLOD1* is a potential therapeutic target for the treatment of mesenchymal gliomas (GBM). PLOD1 also enhances tumor vitality, activity, and migration, and promotes the transformation of GBM into malignant mesenchymal subtypes. They also found that PLOD1 expression is closely related to NF-κB signaling (Wang Z. et al., 2021). Furthermore, high expression level of *PLOD1* was significantly correlated with the degree of response in hepatocellular carcinoma (HCC) patients and was positively correlated with immune infiltration. Further study of *PLOD1* may provide new insights into diagnostic and prognostic biomarkers in HCC patients (Yang et al., 2020).

*PLOD2* encodes lysine hydroxylase 2 (LH2). It is primarily located in the rough endoplasmic reticulum in multiple human tissues. PLOD2 specifically hydroxylates the lysine of the procollagen terminal peptide, which is important for the covalent crosslinking of collagen and tissue stiffness. Therefore, *PLOD2* is a hydroxylase that mainly affects covalent crosslinking of collagen. After being secreted from the cell, the lysine residues of the PLOD2-modified collagen terminal peptide can be crosslinked to form a stable hydroxyl lysine pyridine chain (Noda et al., 2012; Cheriyamundath et al., 2021). In contrast, the crosslinks formed by collagen that has not undergone hydroxylation are less stable and prone to degradation. Therefore, the extent of lysine hydroxylation in the single-chain terminal peptide region of procollagen—that is, the expression and activity of LH2—determines the strength and arrangement of collagen fibers in the ECM (Gilkes et al., 2013; Du et al., 2017). In a variety of tumors, fibrous collagen is considered to provide a channel for cancer cell migration and is mainly modified by PLOD2. For example, PLOD2 can increase the invasiveness and migration of gastric cancer (GC) cells and promote their resistance to 5-fluorouracil by upregulating BCRP and inhibiting apoptosis (Wang et al., 2020).

PLOD3, also known as LH3, has a molecular weight of 85 kDa and catalyzes the hydroxylation of lysine residues in collagen. Studies have shown that PLOD3 has galactose hydroxylysine glucosyltransferase activity, which adds glucose groups to the galactose hydroxylysine residues of collagen (Shao et al., 2020). As previously reported, PLOD3 has a remarkable effect on the synthesis and modification of collagen. As modification of collagen and the extracellular matrix is related to tumor invasion and angiogenesis (Taler et al., 2020), PLOD3 may be involved in tumor development. Guo et al. found that *PLOD3* is associated with ovarian cancer tumor progression and demonstrated that the interaction of COLGALT2 and PLOD3 can enhance the invasiveness of ovarian cancer (Guo et al., 2021b).

At present, *PLODs*, as novel oncogenes, have not been studied in LUAD in detail. Through data exploration, we discovered that *PLODs* were overexpressed in the tumor tissues of LUAD patients. The key findings were confirmed using the PrognoScan, GEPIA, HPA, and CPTAC databases. These results demonstrate that high *PLOD* expression may contribute to advanced disease, poor differentiation, or poor prognosis. Despite the fact that there was little statistical difference between groups, *PLOD* overexpression may be associated with tumor progression.

The more advanced T and N classifications were associated with higher PLOD expression, even though there was no statistically significant intergroup difference, possibly due to insufficient sample content. By analyzing GO, KEGG, and PPI network enrichment, we found that *PLOD1* expression was positively associated with protein digestion and absorption, ECM–receptor interaction, and the AGE-RAGE signaling pathway in diabetic complications. The ECM–receptor interaction pathway mainly destroys the extracellular matrix, one of the necessary conditions for tumor invasion and metastasis. These data provide another explanation for the cancer-promoting mechanism of PLOD1. Functional annotations of *PLOD2* and *PLOD3* indicated that they are involved in protein digestion and absorption, amoebiasis, and AGE-RAGE signaling pathways in diabetic complications (Ruiz et al., 2021). Advanced glycation end products (AGEs) result from reducing sugars and substances, ingredients, or non-enzymatic reactions. AGEs interact with their receptor RAGE to activate multiple signal transduction pathways, as well as to disturb redox balance and regulate multiple cell death pathways (Waghela et al., 2021). Thus, *PLOD* genes play a special role in the progression of LUAD.

The interactions between the immune system and cancer are complex (Symmans, 2021). Several recent studies have revealed that systematic evaluation of immune infiltration is important for predicting clinical outcomes and developing immunotherapies (Nebot-Bral et al., 2017). Immune regulatory cells inhibit the antitumor response; they recognize and eliminate tumor cells that have undergone mutations, thereby inhibiting tumor growth. In addition, the immune system–mediated inflammatory response can promote tumor growth (Wang H. et al., 2021; Hoteit et al., 2021). The alteration of T cell and NK cell compartments found in stage I LUAD lesions suggests that the differential distribution of immune cells is important in the development of immunotherapy strategies for lung cancer (Lavin et al., 2017). The multiple roles of cancer-related T cells in lung cancer have been extensively studied. The characteristic genes of CD8^+^ depletion and pre-depletion T cells, as well as activated tumor Tregs, can be used as clinical biomarkers for LUAD patients (Guo et al., 2018). Studies have pointed out that the expression levels of *PLOD1-3* in hepatocellular carcinoma are positively correlated with the activity of infiltrating immune cells, including macrophages, neutrophils, CD4^+^ T cells, and dendritic cells (Yang et al., 2020). We investigated the relationship between *PLODs* and infiltrating immune cells in LUAD. We found that *PLOD1* was negatively correlated with the abundance of CD8^+^ T cells, cytotoxic cells, and Th cells. It was concluded that the expression of *PLOD1* mainly reduces the infiltration of CD8^+^ T cells, cytotoxic cells, and Th cells. *PLOD2* expression was negatively associated with the abundance of CD8^+^ T cells, B cells, and DCs. *PLOD3* expression was negatively correlated with the abundance of CD8^+^ T cells, B cells, DCs, and cytotoxic cells. In contrast, our research has found that the expression of *PLODs* is positively correlated with the expression of immunosuppressive cells, such as Tregs and MDSCs. Based on our immunological analysis, we hypothesize that *PLODs* play an important role in immune escape in patients with LUAD.

In summary, we first proposed that *PLOD* expression is upregulated in LUAD. Our findings underscore the importance of *PLODs* in relation to disease stage, degree of differentiation, tumor size, and lymph-node metastasis. Therefore, *PLODs* can be considered an early diagnosis and independent prognostic biomarker for patients with LUAD. Compared with traditional experimental analysis, analyses using tumor databases have the advantages of larger sample sizes and higher reliability, which also provide a preliminary foundation for further research. Our data provide new potential indicators for the clinical prediction of patient survival and fundamental support for the progress of immunotherapy targets. This study provides a new strategy for individualized immunotherapy of LUAD, which will benefit more patients. In future studies, we will further verify the value of PLOD in LUAD.

## Data Availability

The datasets presented in this study can be found in online repositories. The names of the repository/repositories and accession number(s) can be found in the article/Supplementary Material.
