# Supplementary material for: Clinical Prognostic Value of the PLOD Gene Family in Lung Adenocarcinoma
Source: Front Mol Biosci. 2022 Feb 21;8:770729. doi: 10.3389/fmolb.2021.770729 (PMC8899219; doi:10.3389/fmolb.2021.770729)
Supplement: Supplementary file 1 [file Table1.docx]

**sTable.1 Expression pattern of PLOD1 in Pan-cancer perspective.**

| **Tumor** | **Type** | **Number** | **Max** | **Minimum** | **Median** | **IQR** | **Lower quartile** | **Upper quartile** | | **Mean** | | **SD** | | **SE** | |
| --- | --- | --- | --- | --- | --- | --- | --- | --- | --- | --- | --- | --- | --- | --- | --- |
| ACC | Tumor | 79 | 2.8 | 8.745 | 5.882 | 1.246 | 5.216 | 6.462 | 5.867 | | 1.122 | | 0.126 | |  |
| BLCA | Normal | 19 | 4.568 | 6.749 | 5.805 | 0.625 | 5.433 | 6.058 | 5.729 | | 0.604 | | 0.139 | |  |
| BLCA | Tumor | 414 | 3.041 | 9.49 | 6.563 | 1.063 | 6.038 | 7.101 | 6.572 | | 0.885 | | 0.044 | |  |
| BRCA | Normal | 113 | 4.231 | 7.56 | 6.287 | 0.494 | 6.074 | 6.568 | 6.271 | | 0.475 | | 0.045 | |  |
| BRCA | Tumor | 1109 | 3.173 | 10.352 | 6.604 | 0.783 | 6.265 | 7.048 | 6.701 | | 0.811 | | 0.024 | |  |
| CESC | Normal | 3 | 6.493 | 6.951 | 6.537 | 0.229 | 6.515 | 6.744 | 6.66 | | 0.253 | | 0.146 | |  |
| CESC | Tumor | 306 | 3.601 | 9.602 | 6.497 | 1.02 | 6.02 | 7.041 | 6.537 | | 0.84 | | 0.048 | |  |
| CHOL | Normal | 9 | 5.444 | 6.221 | 5.787 | 0.395 | 5.509 | 5.904 | 5.759 | | 0.254 | | 0.085 | |  |
| CHOL | Tumor | 36 | 4.349 | 8.856 | 6.027 | 1.093 | 5.599 | 6.692 | 6.095 | | 0.93 | | 0.155 | |  |
| COAD | Normal | 41 | 4.306 | 6.763 | 5.346 | 0.454 | 5.076 | 5.53 | 5.368 | | 0.488 | | 0.076 | |  |
| COAD | Tumor | 480 | 2.463 | 8.263 | 6.248 | 0.81 | 5.816 | 6.625 | 6.191 | | 0.71 | | 0.032 | |  |
| DLBC | Tumor | 48 | 4.593 | 7.096 | 5.616 | 0.619 | 5.38 | 6 | 5.635 | | 0.546 | | 0.079 | |  |
| ESCA | Normal | 11 | 3.738 | 6.6 | 4.707 | 1.285 | 4.171 | 5.455 | 4.911 | | 0.97 | | 0.292 | |  |
| ESCA | Tumor | 162 | 3.738 | 8.384 | 6.311 | 1.069 | 5.8 | 6.869 | 6.308 | | 0.834 | | 0.065 | |  |
| GBM | Normal | 5 | 4.713 | 5.951 | 4.873 | 0.265 | 4.808 | 5.073 | 5.084 | | 0.503 | | 0.225 | |  |
| GBM | Tumor | 169 | 5.145 | 9.196 | 6.979 | 0.882 | 6.495 | 7.377 | 6.955 | | 0.714 | | 0.055 | |  |
| HNSC | Normal | 44 | 3.488 | 6.872 | 4.694 | 0.804 | 4.363 | 5.167 | 4.859 | | 0.778 | | 0.117 | |  |
| HNSC | Tumor | 502 | 4.054 | 9.396 | 6.726 | 1.125 | 6.181 | 7.305 | 6.747 | | 0.843 | | 0.038 | |  |
| KICH | Normal | 24 | 5.074 | 6.882 | 5.725 | 0.65 | 5.595 | 6.245 | 5.918 | | 0.473 | | 0.097 | |  |
| KICH | Tumor | 65 | 2.551 | 8.596 | 4.334 | 0.741 | 3.922 | 4.664 | 4.374 | | 1.012 | | 0.126 | |  |
| KIRC | Normal | 72 | 5.168 | 7.936 | 5.85 | 0.518 | 5.664 | 6.183 | 5.914 | | 0.422 | | 0.05 | |  |
| KIRC | Tumor | 539 | 1.934 | 10.359 | 7.232 | 0.84 | 6.816 | 7.656 | 7.15 | | 0.932 | | 0.04 | |  |
| KIRP | Normal | 32 | 5.073 | 7.158 | 6.039 | 0.682 | 5.809 | 6.491 | 6.117 | | 0.489 | | 0.086 | |  |
| KIRP | Tumor | 289 | 3.763 | 8.818 | 6.174 | 0.891 | 5.645 | 6.537 | 6.138 | | 0.769 | | 0.045 | |  |
| LAML | Tumor | 151 | 2.171 | 6.769 | 4.965 | 1.071 | 4.387 | 5.458 | 4.931 | | 0.786 | | 0.064 | |  |
| LGG | Tumor | 529 | 3.127 | 8.303 | 5.615 | 0.943 | 5.144 | 6.087 | 5.66 | | 0.689 | | 0.03 | |  |
| LIHC | Normal | 50 | 5.103 | 6.396 | 5.828 | 0.536 | 5.569 | 6.105 | 5.827 | | 0.315 | | 0.045 | |  |
| LIHC | Tumor | 374 | 4.718 | 9.681 | 6.658 | 0.991 | 6.148 | 7.139 | 6.695 | | 0.788 | | 0.041 | |  |
| LUAD | Normal | 59 | 5.328 | 6.811 | 5.837 | 0.342 | 5.694 | 6.036 | 5.89 | | 0.294 | | 0.038 | |  |
| LUAD | Tumor | 535 | 3.503 | 9.132 | 6.628 | 0.809 | 6.205 | 7.015 | 6.602 | | 0.738 | | 0.032 | |  |
| LUSC | Normal | 49 | 5.169 | 7.495 | 6.025 | 0.486 | 5.818 | 6.304 | 6.092 | | 0.47 | | 0.067 | |  |
| LUSC | Tumor | 502 | 4.784 | 10.136 | 6.98 | 1.109 | 6.472 | 7.581 | 7.052 | | 0.863 | | 0.038 | |  |
| MESO | Tumor | 86 | 4.716 | 9.44 | 6.954 | 1.157 | 6.374 | 7.531 | 6.958 | | 0.933 | | 0.101 | |  |
| OV | Tumor | 379 | 3.747 | 9.233 | 6.204 | 1.013 | 5.733 | 6.746 | 6.221 | | 0.79 | | 0.041 | |  |
| PAAD | Normal | 4 | 4.665 | 7.346 | 6.869 | 0.784 | 6.261 | 7.046 | 6.437 | | 1.204 | | 0.602 | |  |
| PAAD | Tumor | 178 | 4.334 | 8.897 | 6.553 | 0.867 | 6.125 | 6.992 | 6.572 | | 0.81 | | 0.061 | |  |
| PCPG | Normal | 3 | 5.052 | 5.643 | 5.191 | 0.296 | 5.122 | 5.417 | 5.295 | | 0.309 | | 0.178 | |  |
| PCPG | Tumor | 183 | 2.691 | 8 | 5.854 | 1.007 | 5.322 | 6.329 | 5.845 | | 0.846 | | 0.063 | |  |
| PRAD | Normal | 52 | 4.927 | 6.937 | 6.364 | 0.517 | 6.108 | 6.624 | 6.279 | | 0.466 | | 0.065 | |  |
| PRAD | Tumor | 499 | 3.311 | 7.745 | 6.436 | 0.52 | 6.172 | 6.692 | 6.4 | | 0.476 | | 0.021 | |  |
| READ | Normal | 10 | 4.732 | 5.879 | 5.501 | 0.348 | 5.311 | 5.66 | 5.453 | | 0.351 | | 0.111 | |  |
| READ | Tumor | 167 | 4.783 | 7.94 | 6.152 | 0.614 | 5.859 | 6.473 | 6.187 | | 0.532 | | 0.041 | |  |
| SARC | Normal | 2 | 6.213 | 6.895 | 6.554 | 0.341 | 6.384 | 6.725 | 6.554 | | 0.482 | | 0.341 | |  |
| SARC | Tumor | 263 | 4.66 | 10.383 | 7.987 | 1.448 | 7.288 | 8.737 | 7.994 | | 0.99 | | 0.061 | |  |
| SKCM | Normal | 1 | 7.198 | 7.198 | 7.198 | 0 | 7.198 | 7.198 | 7.198 | |  | |  | |  |
| SKCM | Tumor | 471 | 4.651 | 10.3 | 7.794 | 1.081 | 7.191 | 8.273 | 7.747 | | 0.843 | | 0.039 | |  |
| STAD | Normal | 32 | 3.555 | 6.612 | 5.208 | 0.998 | 4.6 | 5.597 | 5.122 | | 0.799 | | 0.141 | |  |
| STAD | Tumor | 375 | 4.095 | 8.139 | 6.169 | 0.919 | 5.687 | 6.606 | 6.138 | | 0.695 | | 0.036 | |  |
| TGCT | Tumor | 156 | 5.449 | 8.998 | 7.316 | 0.831 | 6.933 | 7.764 | 7.325 | | 0.616 | | 0.049 | |  |
| THCA | Normal | 58 | 4.76 | 6.533 | 6.089 | 0.381 | 5.895 | 6.276 | 6.064 | | 0.295 | | 0.039 | |  |
| THCA | Tumor | 510 | 4.459 | 8.505 | 6.298 | 0.596 | 6.016 | 6.612 | 6.327 | | 0.535 | | 0.024 | |  |
| THYM | Normal | 2 | 3.942 | 6.175 | 5.059 | 1.117 | 4.501 | 5.617 | 5.059 | | 1.579 | | 1.117 | |  |
| THYM | Tumor | 119 | 2.963 | 7.472 | 5.018 | 1.633 | 4.248 | 5.881 | 5.107 | | 1.026 | | 0.094 | |  |
| UCEC | Normal | 35 | 5.739 | 8.369 | 6.756 | 0.713 | 6.312 | 7.025 | 6.763 | | 0.644 | | 0.109 | |  |
| UCEC | Tumor | 552 | 4.766 | 9.967 | 7.629 | 0.975 | 7.117 | 8.092 | 7.581 | | 0.783 | | 0.033 | |  |
| UCS | Tumor | 56 | 6.454 | 9.448 | 8.014 | 0.929 | 7.663 | 8.592 | 8.072 | | 0.683 | | 0.091 | |  |
| UVM | Tumor | 80 | 5.002 | 8.077 | 6.997 | 0.812 | 6.568 | 7.38 | 6.919 | | 0.693 | | 0.077 | |  |
