# Supplementary figures and images for: Clinical Prognostic Value of the PLOD Gene Family in Lung Adenocarcinoma
Source: Front Mol Biosci. 2022 Feb 21;8:770729. doi: 10.3389/fmolb.2021.770729 (PMC8899219; doi:10.3389/fmolb.2021.770729)

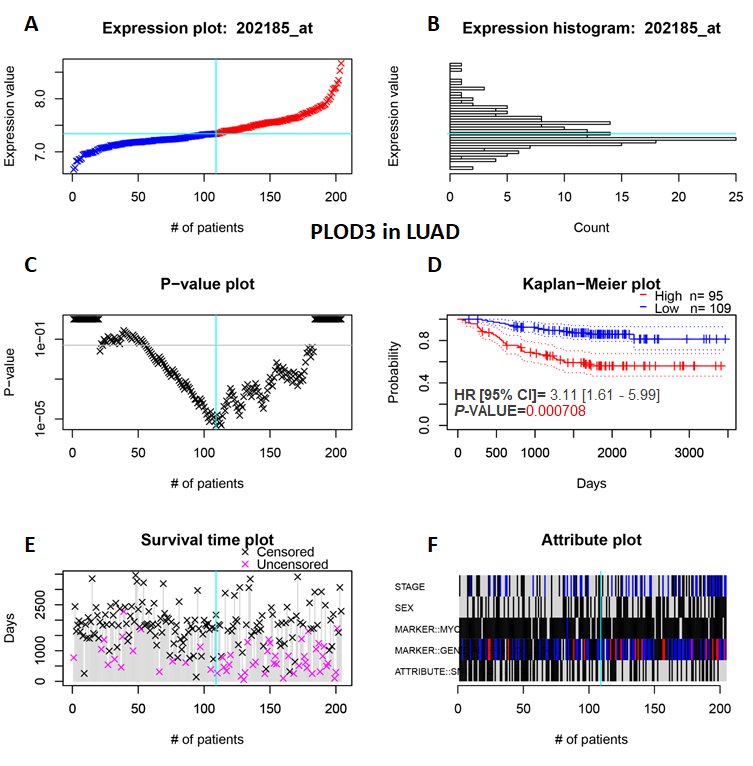

Supplement: Supplementary file 2 [file Image3.TIF]

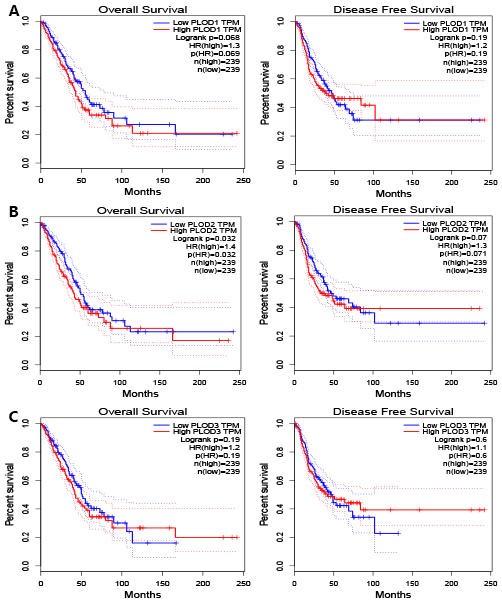

Supplement: Supplementary file 3 [file Image4.TIF]

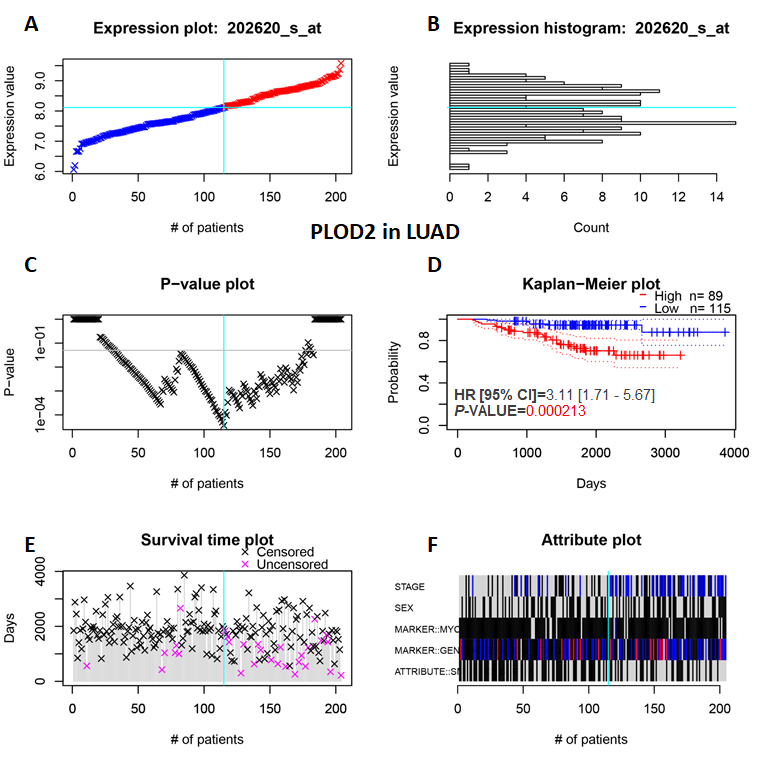

Supplement: Supplementary file 4 [file Image2.TIF]

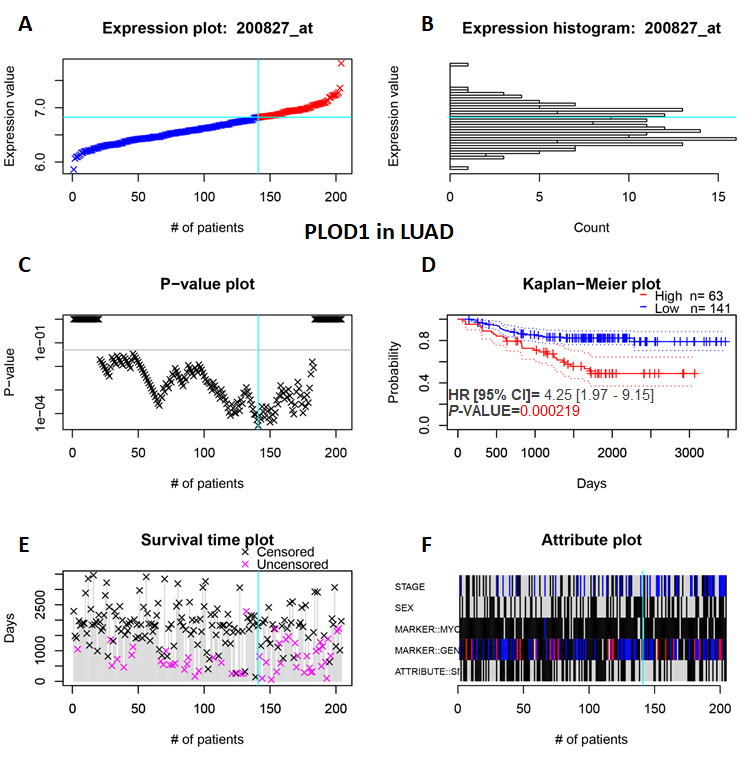

Supplement: Supplementary file 5 [file Image1.TIF]
