# Supplementary material for: Clinical Prognostic Value of the PLOD Gene Family in Lung Adenocarcinoma
Source: Front Mol Biosci. 2022 Feb 21;8:770729. doi: 10.3389/fmolb.2021.770729 (PMC8899219; doi:10.3389/fmolb.2021.770729)
Supplement: Supplementary file 6 [file Table2.docx]

**sTable.2 Expression pattern of PLOD2 in Pan-cancer perspective.**

| **Tumor** | **Type** | **Number** | **Max** | **Minimum** | **Median** | **IQR** | **Lower quartile** | **Upper quartile** | **Mean** | **SD** | **SE** |
| --- | --- | --- | --- | --- | --- | --- | --- | --- | --- | --- | --- |
| ACC | Tumor | 79 | 1.117 | 7.745 | 4.008 | 1.741 | 2.915 | 4.657 | 3.903 | 1.359 | 0.153 |
| BLCA | Normal | 19 | 2.408 | 6.434 | 3.844 | 1.888 | 2.93 | 4.819 | 3.952 | 1.203 | 0.276 |
| BLCA | Tumor | 414 | 0.489 | 9.324 | 4.523 | 1.986 | 3.402 | 5.387 | 4.398 | 1.429 | 0.07 |
| BRCA | Normal | 113 | 1.948 | 7.456 | 4.685 | 1.485 | 4.178 | 5.663 | 4.853 | 0.93 | 0.088 |
| BRCA | Tumor | 1109 | 0.375 | 9.77 | 4.837 | 1.527 | 4.069 | 5.596 | 4.803 | 1.162 | 0.035 |
| CESC | Normal | 3 | 4.231 | 5.779 | 4.474 | 0.774 | 4.353 | 5.127 | 4.828 | 0.833 | 0.481 |
| CESC | Tumor | 306 | 1.158 | 8.709 | 5.52 | 1.399 | 4.799 | 6.198 | 5.527 | 1.231 | 0.07 |
| CHOL | Normal | 9 | 3.707 | 6.041 | 4.011 | 0.362 | 3.788 | 4.149 | 4.204 | 0.718 | 0.239 |
| CHOL | Tumor | 36 | 1.03 | 7.041 | 4.104 | 2.126 | 2.6 | 4.726 | 3.804 | 1.548 | 0.258 |
| COAD | Normal | 41 | 4.419 | 6.646 | 5.448 | 0.964 | 4.942 | 5.906 | 5.467 | 0.621 | 0.097 |
| COAD | Tumor | 480 | 1.421 | 7.33 | 4.703 | 1.253 | 4.024 | 5.277 | 4.612 | 1.058 | 0.048 |
| DLBC | Tumor | 48 | 0.481 | 4.576 | 2.348 | 1.471 | 1.544 | 3.015 | 2.346 | 1.08 | 0.156 |
| ESCA | Normal | 11 | 2.017 | 5.705 | 3.872 | 0.852 | 3.3 | 4.151 | 3.787 | 0.965 | 0.291 |
| ESCA | Tumor | 162 | 2.181 | 8.047 | 4.785 | 1.861 | 3.844 | 5.705 | 4.77 | 1.229 | 0.097 |
| GBM | Normal | 5 | 3.032 | 3.727 | 3.256 | 0.455 | 3.228 | 3.684 | 3.385 | 0.305 | 0.136 |
| GBM | Tumor | 169 | 2.93 | 7.747 | 5.397 | 1.054 | 4.897 | 5.951 | 5.402 | 0.918 | 0.071 |
| HNSC | Normal | 44 | 1.377 | 4.572 | 3.318 | 0.824 | 2.82 | 3.645 | 3.183 | 0.708 | 0.107 |
| HNSC | Tumor | 502 | 0.629 | 7.869 | 4.891 | 1.596 | 4.07 | 5.666 | 4.795 | 1.24 | 0.055 |
| KICH | Normal | 24 | 4.147 | 5.971 | 5.316 | 0.794 | 4.88 | 5.674 | 5.264 | 0.507 | 0.103 |
| KICH | Tumor | 65 | 1.972 | 7.745 | 4.254 | 0.984 | 3.8 | 4.784 | 4.318 | 0.989 | 0.123 |
| KIRC | Normal | 72 | 3.49 | 7.868 | 4.987 | 1.219 | 4.5 | 5.719 | 5.079 | 0.828 | 0.098 |
| KIRC | Tumor | 539 | 2.1 | 11.058 | 6.624 | 1.241 | 6.048 | 7.289 | 6.634 | 1.131 | 0.049 |
| KIRP | Normal | 32 | 3.539 | 5.931 | 4.495 | 1.07 | 3.989 | 5.059 | 4.541 | 0.649 | 0.115 |
| KIRP | Tumor | 289 | 1.514 | 8.849 | 5.479 | 1.374 | 4.725 | 6.099 | 5.418 | 1.057 | 0.062 |
| LAML | Tumor | 151 | 0 | 4.503 | 0.127 | 0.3 | 0.038 | 0.338 | 0.354 | 0.683 | 0.056 |
| LGG | Tumor | 529 | 0.683 | 6.599 | 4.013 | 1.065 | 3.414 | 4.479 | 3.965 | 0.895 | 0.039 |
| LIHC | Normal | 50 | 2.589 | 5.502 | 4.052 | 0.652 | 3.838 | 4.49 | 4.137 | 0.587 | 0.083 |
| LIHC | Tumor | 374 | 0.669 | 8.182 | 4.635 | 1.501 | 3.908 | 5.409 | 4.601 | 1.127 | 0.058 |
| LUAD | Normal | 59 | 2.619 | 4.469 | 3.793 | 0.418 | 3.53 | 3.948 | 3.726 | 0.375 | 0.049 |
| LUAD | Tumor | 535 | 1.267 | 9.165 | 5.112 | 2.033 | 4.025 | 6.058 | 5.091 | 1.406 | 0.061 |
| LUSC | Normal | 49 | 3.065 | 5.357 | 3.796 | 0.558 | 3.484 | 4.042 | 3.836 | 0.505 | 0.072 |
| LUSC | Tumor | 502 | 2.292 | 9.107 | 5.627 | 1.487 | 4.94 | 6.427 | 5.639 | 1.06 | 0.047 |
| MESO | Tumor | 86 | 3.364 | 9.051 | 7.389 | 1.235 | 6.638 | 7.873 | 7.162 | 1.197 | 0.129 |
| OV | Tumor | 379 | 1.549 | 7.527 | 4.795 | 1.403 | 4.112 | 5.515 | 4.799 | 1.037 | 0.053 |
| PAAD | Normal | 4 | 2.658 | 5.755 | 5.737 | 0.775 | 4.967 | 5.742 | 4.972 | 1.543 | 0.771 |
| PAAD | Tumor | 178 | 0.288 | 7.954 | 4.86 | 1.345 | 4.229 | 5.573 | 4.83 | 1.143 | 0.086 |
| PCPG | Normal | 3 | 3.605 | 4.792 | 4.077 | 0.593 | 3.841 | 4.434 | 4.158 | 0.597 | 0.345 |
| PCPG | Tumor | 183 | 1.39 | 6.368 | 3.425 | 0.947 | 3.021 | 3.969 | 3.485 | 0.754 | 0.056 |
| PRAD | Normal | 52 | 2.521 | 4.884 | 3.85 | 0.74 | 3.351 | 4.091 | 3.76 | 0.567 | 0.079 |
| PRAD | Tumor | 499 | 0.566 | 6.255 | 3.224 | 1.171 | 2.675 | 3.846 | 3.25 | 0.916 | 0.041 |
| READ | Normal | 10 | 4.44 | 6.675 | 5.198 | 0.533 | 5.101 | 5.633 | 5.435 | 0.677 | 0.214 |
| READ | Tumor | 167 | 1.627 | 7.246 | 4.696 | 1.386 | 4.003 | 5.389 | 4.638 | 1.034 | 0.08 |
| SARC | Normal | 2 | 4.11 | 4.67 | 4.39 | 0.28 | 4.25 | 4.53 | 4.39 | 0.396 | 0.28 |
| SARC | Tumor | 263 | 1.46 | 8.868 | 5.538 | 1.94 | 4.612 | 6.553 | 5.582 | 1.316 | 0.081 |
| SKCM | Normal | 1 | 3.657 | 3.657 | 3.657 | 0 | 3.657 | 3.657 | 3.657 |  |  |
| SKCM | Tumor | 471 | 0.293 | 7.389 | 4.09 | 1.91 | 3.048 | 4.958 | 3.949 | 1.401 | 0.065 |
| STAD | Normal | 32 | 2.148 | 5.133 | 3.411 | 1.324 | 2.87 | 4.194 | 3.575 | 0.858 | 0.152 |
| STAD | Tumor | 375 | 1.357 | 6.465 | 4.211 | 1.331 | 3.546 | 4.876 | 4.187 | 0.975 | 0.05 |
| TGCT | Tumor | 156 | 2.208 | 7.029 | 4.764 | 1.029 | 4.277 | 5.306 | 4.775 | 0.8 | 0.064 |
| THCA | Normal | 58 | 4.239 | 7.276 | 6.564 | 0.536 | 6.276 | 6.812 | 6.462 | 0.56 | 0.074 |
| THCA | Tumor | 510 | 2.541 | 8.18 | 6.66 | 0.828 | 6.204 | 7.033 | 6.58 | 0.675 | 0.03 |
| THYM | Normal | 2 | 3.07 | 4.351 | 3.71 | 0.64 | 3.39 | 4.03 | 3.71 | 0.906 | 0.64 |
| THYM | Tumor | 119 | 0.783 | 7.12 | 4.241 | 1.616 | 3.37 | 4.985 | 4.171 | 1.099 | 0.101 |
| UCEC | Normal | 35 | 2.738 | 5.348 | 4.297 | 0.607 | 4.014 | 4.621 | 4.281 | 0.581 | 0.098 |
| UCEC | Tumor | 552 | 0.939 | 7.626 | 4.759 | 1.721 | 3.817 | 5.539 | 4.63 | 1.33 | 0.057 |
| UCS | Tumor | 56 | 2.856 | 7.715 | 5.519 | 1.326 | 4.678 | 6.004 | 5.449 | 1.048 | 0.14 |
| UVM | Tumor | 80 | 0.157 | 3.915 | 1.511 | 1.468 | 0.973 | 2.441 | 1.699 | 0.948 | 0.106 |
