# Supplementary material for: Clinical Prognostic Value of the PLOD Gene Family in Lung Adenocarcinoma
Source: Front Mol Biosci. 2022 Feb 21;8:770729. doi: 10.3389/fmolb.2021.770729 (PMC8899219; doi:10.3389/fmolb.2021.770729)
Supplement: Supplementary file 7 [file Table3.docx]

**sTable.3 Expression pattern of PLOD3 in Pan-cancer perspective.**

| **Tumor** | **Type** | **Number** | **Max** | **Minimum** | **Median** | **IQR** | **Lower quartile** | **Upper quartile** | **Mean** | **SD** | **SE** |
| --- | --- | --- | --- | --- | --- | --- | --- | --- | --- | --- | --- |
| ACC | Tumor | 79 | 2.424 | 8.031 | 5.723 | 1.374 | 5.174 | 6.548 | 5.76 | 0.993 | 0.112 |
| BLCA | Normal | 19 | 2.823 | 5.52 | 4.994 | 0.632 | 4.584 | 5.216 | 4.781 | 0.703 | 0.161 |
| BLCA | Tumor | 414 | 2.65 | 8.461 | 5.482 | 1.004 | 4.962 | 5.967 | 5.481 | 0.783 | 0.038 |
| BRCA | Normal | 113 | 2.451 | 5.752 | 4.89 | 0.439 | 4.655 | 5.095 | 4.835 | 0.435 | 0.041 |
| BRCA | Tumor | 1109 | 1.526 | 8.511 | 5.39 | 0.703 | 5.028 | 5.731 | 5.421 | 0.746 | 0.022 |
| CESC | Normal | 3 | 5.196 | 6.027 | 5.619 | 0.416 | 5.408 | 5.823 | 5.614 | 0.416 | 0.24 |
| CESC | Tumor | 306 | 1.116 | 7.938 | 4.923 | 1.079 | 4.399 | 5.478 | 4.904 | 0.881 | 0.05 |
| CHOL | Normal | 9 | 3.907 | 4.429 | 4.209 | 0.419 | 3.945 | 4.364 | 4.17 | 0.207 | 0.069 |
| CHOL | Tumor | 36 | 4.566 | 7.845 | 6.665 | 0.828 | 6.126 | 6.954 | 6.548 | 0.658 | 0.11 |
| COAD | Normal | 41 | 4.844 | 6.021 | 5.533 | 0.373 | 5.283 | 5.656 | 5.481 | 0.248 | 0.039 |
| COAD | Tumor | 480 | 2.301 | 8.657 | 6.747 | 0.82 | 6.353 | 7.173 | 6.696 | 0.828 | 0.038 |
| DLBC | Tumor | 48 | 3.869 | 6.393 | 4.819 | 0.631 | 4.5 | 5.132 | 4.829 | 0.516 | 0.074 |
| ESCA | Normal | 11 | 2.509 | 5.572 | 4.212 | 0.85 | 3.752 | 4.601 | 4.203 | 0.858 | 0.259 |
| ESCA | Tumor | 162 | 2.08 | 8.208 | 5.899 | 1.458 | 5.111 | 6.569 | 5.817 | 1.071 | 0.084 |
| GBM | Normal | 5 | 3.364 | 6.436 | 4.041 | 0.604 | 3.522 | 4.127 | 4.298 | 1.239 | 0.554 |
| GBM | Tumor | 169 | 3.83 | 8.596 | 6.196 | 0.914 | 5.74 | 6.654 | 6.176 | 0.699 | 0.054 |
| HNSC | Normal | 44 | 1.332 | 4.876 | 3.198 | 0.839 | 2.892 | 3.73 | 3.351 | 0.771 | 0.116 |
| HNSC | Tumor | 502 | 2.272 | 8.093 | 5.398 | 1.209 | 4.733 | 5.942 | 5.364 | 0.936 | 0.042 |
| KICH | Normal | 24 | 3.984 | 6.041 | 4.815 | 0.546 | 4.561 | 5.107 | 4.886 | 0.453 | 0.093 |
| KICH | Tumor | 65 | 3.202 | 7.785 | 4.915 | 0.57 | 4.605 | 5.174 | 4.91 | 0.722 | 0.09 |
| KIRC | Normal | 72 | 3.758 | 6.565 | 4.545 | 0.501 | 4.352 | 4.853 | 4.591 | 0.461 | 0.054 |
| KIRC | Tumor | 539 | 2.796 | 8.937 | 5.927 | 0.588 | 5.67 | 6.258 | 5.93 | 0.668 | 0.029 |
| KIRP | Normal | 32 | 4.287 | 5.945 | 4.818 | 0.309 | 4.688 | 4.997 | 4.892 | 0.37 | 0.065 |
| KIRP | Tumor | 289 | 3.95 | 8.003 | 6.166 | 1.043 | 5.592 | 6.635 | 6.109 | 0.759 | 0.045 |
| LAML | Tumor | 151 | 2.911 | 6.696 | 4.898 | 0.915 | 4.384 | 5.298 | 4.855 | 0.742 | 0.06 |
| LGG | Tumor | 529 | 3.062 | 7.705 | 5.082 | 0.782 | 4.72 | 5.502 | 5.145 | 0.673 | 0.029 |
| LIHC | Normal | 50 | 3.227 | 5.411 | 4.207 | 0.575 | 3.879 | 4.454 | 4.203 | 0.491 | 0.069 |
| LIHC | Tumor | 374 | 4.04 | 8.477 | 5.826 | 1.053 | 5.292 | 6.344 | 5.82 | 0.738 | 0.038 |
| LUAD | Normal | 59 | 4.245 | 5.891 | 5.235 | 0.315 | 5.081 | 5.396 | 5.241 | 0.285 | 0.037 |
| LUAD | Tumor | 535 | 2.621 | 7.991 | 5.553 | 0.733 | 5.192 | 5.925 | 5.531 | 0.72 | 0.031 |
| LUSC | Normal | 49 | 4.329 | 6.147 | 5.327 | 0.347 | 5.19 | 5.537 | 5.321 | 0.346 | 0.049 |
| LUSC | Tumor | 502 | 2.662 | 8.19 | 5.686 | 1 | 5.28 | 6.28 | 5.735 | 0.791 | 0.035 |
| MESO | Tumor | 86 | 5.024 | 7.678 | 6.162 | 0.796 | 5.863 | 6.66 | 6.235 | 0.564 | 0.061 |
| OV | Tumor | 379 | 3.719 | 6.895 | 5.305 | 0.696 | 4.929 | 5.625 | 5.275 | 0.562 | 0.029 |
| PAAD | Normal | 4 | 4.686 | 6.202 | 5.777 | 0.67 | 5.359 | 6.029 | 5.611 | 0.667 | 0.334 |
| PAAD | Tumor | 178 | 3.414 | 8.136 | 6.086 | 0.607 | 5.749 | 6.357 | 6.022 | 0.637 | 0.048 |
| PCPG | Normal | 3 | 4.243 | 4.656 | 4.318 | 0.207 | 4.28 | 4.487 | 4.406 | 0.22 | 0.127 |
| PCPG | Tumor | 183 | 3.554 | 7.694 | 5.837 | 0.762 | 5.432 | 6.194 | 5.779 | 0.667 | 0.049 |
| PRAD | Normal | 52 | 3.774 | 5.911 | 4.884 | 0.49 | 4.573 | 5.063 | 4.842 | 0.404 | 0.056 |
| PRAD | Tumor | 499 | 1.864 | 6.538 | 5.155 | 0.595 | 4.875 | 5.47 | 5.155 | 0.519 | 0.023 |
| READ | Normal | 10 | 4.641 | 5.87 | 5.403 | 0.488 | 5.164 | 5.653 | 5.335 | 0.427 | 0.135 |
| READ | Tumor | 167 | 4.93 | 8.837 | 6.909 | 0.684 | 6.562 | 7.246 | 6.899 | 0.614 | 0.047 |
| SARC | Normal | 2 | 4.553 | 5.4 | 4.977 | 0.424 | 4.765 | 5.188 | 4.977 | 0.599 | 0.424 |
| SARC | Tumor | 263 | 4.226 | 9.463 | 6.477 | 0.971 | 6.039 | 7.01 | 6.527 | 0.739 | 0.046 |
| SKCM | Normal | 1 | 7.004 | 7.004 | 7.004 | 0 | 7.004 | 7.004 | 7.004 |  |  |
| SKCM | Tumor | 471 | 4.668 | 10.548 | 8.13 | 1.101 | 7.596 | 8.697 | 8.11 | 0.891 | 0.041 |
| STAD | Normal | 32 | 1.982 | 5.3 | 4.294 | 0.983 | 3.707 | 4.691 | 4.157 | 0.809 | 0.143 |
| STAD | Tumor | 375 | 2.654 | 9.089 | 6.087 | 1.128 | 5.365 | 6.493 | 5.985 | 0.86 | 0.044 |
| TGCT | Tumor | 156 | 3.586 | 6.89 | 5.812 | 0.54 | 5.551 | 6.092 | 5.773 | 0.535 | 0.043 |
| THCA | Normal | 58 | 4.117 | 5.362 | 4.872 | 0.211 | 4.799 | 5.009 | 4.897 | 0.194 | 0.026 |
| THCA | Tumor | 510 | 2.407 | 6.9 | 5.092 | 0.46 | 4.833 | 5.294 | 5.072 | 0.394 | 0.017 |
| THYM | Normal | 2 | 4.146 | 5.234 | 4.69 | 0.544 | 4.418 | 4.962 | 4.69 | 0.77 | 0.544 |
| THYM | Tumor | 119 | 3.594 | 6.094 | 4.491 | 0.664 | 4.159 | 4.823 | 4.541 | 0.499 | 0.046 |
| UCEC | Normal | 35 | 4.828 | 6.086 | 5.59 | 0.455 | 5.237 | 5.692 | 5.489 | 0.309 | 0.052 |
| UCEC | Tumor | 552 | 2.625 | 8.343 | 5.693 | 0.852 | 5.256 | 6.108 | 5.663 | 0.718 | 0.031 |
| UCS | Tumor | 56 | 4.629 | 7.561 | 6.001 | 0.962 | 5.679 | 6.641 | 6.154 | 0.686 | 0.092 |
| UVM | Tumor | 80 | 6.029 | 8.603 | 7.554 | 0.782 | 7.123 | 7.905 | 7.499 | 0.602 | 0.067 |
