# Supplementary material for: Clinical Prognostic Value of the PLOD Gene Family in Lung Adenocarcinoma
Source: Front Mol Biosci. 2022 Feb 21;8:770729. doi: 10.3389/fmolb.2021.770729 (PMC8899219; doi:10.3389/fmolb.2021.770729)
Supplement: Supplementary file 8 [file DataSheet1.docx]

**Supplement**

**Figure legend**

**SFigure.1 Analysis of clinical prognosis with *PLOD1*.**

In order to clarify whether the transcription level of *PLOD1* is related to cancer prognosis, we used ProgonoScan to detect its potential role in LUAD. **(A)** Gene expression map, which ranks patients by the expression value of *PLOD1*. The X-axis shows the cumulative number of patients, and the Y-axis shows the expression value. The straight line (cyan) shows the best segmentation point for dividing the patient into high (red) and low (blue) expression groups. **(B)** Expression histogram, showing the distribution of expression values. The X-axis shows the number of patients, the Y-axis shows the expression values, and the cyan line shows the best segmentation point. **(C)** P value graph, for each potential segmentation point of gene expression value. The patients are divided into high expression group and low expression group, and the survival difference is calculated by log-rank test. The X-axis represents the cumulative number of patients, and the Y-axis represents the original P value after logarithm processing. The cyan line represents the cut point where the P value is determined and minimized, and the gray line represents the 5% significance level. **(D)** Kaplan-Meier diagram, plotting the survival curve of the high (red) and low (blue) expression groups bisected by the best split point. The X axis represents time, the Y axis represents survival rate, and the 95% confidence interval of each group is represented by a dotted line. **(E)** Survival time chart, where the X-axis shows the cumulative number of patients. The Y-axis shows the survival time, the black dots represent the censored data, the pink dots represent the uncensored data, and the cyan represents the best segmentation point. **(F)** Attribute distribution map, based on the MARKER and SCORE information provided at the end of the above table.

**SFigure.2 Analysis of clinical prognosis with *PLOD2*.**

**(A)** Gene expression map; **(B)** Expression histogram; **(C)** P value graph; **(D)** Kaplan-Meier diagram; **(E)** Survival time chart; **(F)** Attribute distribution map.

**SFigure.3 Analysis of clinical prognosis with *PLOD3*.**

**(A)** Gene expression map; **(B)** Expression histogram; **(C)** P value graph; **(D)** Kaplan-Meier diagram; **(E)** Survival time chart; **(F)** Attribute distribution map.

**SFigure.4** **Prognostic value of *PLODs***

To gain further insights into the prognostic value of *PLODs* in LUAD, we evaluated the OS and RFS by GEPIA database. The plot was depicted as follow: **(A)** *PLOD1*, **(B)** *PLOD2* and **(C)** *PLOD3*. The results revealed that higher *PLODs* expression were usually associated with poorer prognosis in LUAD patients.
